# Transforming absolute value to categorical choice in primate superior colliculus during value-based decision making

**DOI:** 10.1038/s41467-021-23747-z

**Published:** 2021-06-07

**Authors:** Beizhen Zhang, Janis Ying Ying Kan, Mingpo Yang, Xiaochun Wang, Jiahao Tu, Michael Christopher Dorris

**Affiliations:** 1 Institute of Neuroscience, Key Laboratory of Primate Neurobiology, CAS Center for Excellence in Brain Science and Intelligence Technology, Chinese Academy of Sciences, Beijing, China; 2 University of Chinese Academy of Sciences, Beijing, China; 3 Department of Biomedical and Molecular Sciences, Centre for Neuroscience Studies, Queen’s University, Kingston, ON Canada

**Keywords:** Decision, Superior colliculus, Reward

## Abstract

Value-based decision making involves choosing from multiple options with different values. Despite extensive studies on value representation in various brain regions, the neural mechanism for how multiple value options are converted to motor actions remains unclear. To study this, we developed a multi-value foraging task with varying menu of items in non-human primates using eye movements that dissociates value and choice, and conducted electrophysiological recording in the midbrain superior colliculus (SC). SC neurons encoded “absolute” value, independent of available options, during late fixation. In addition, SC neurons also represent value threshold, modulated by available options, different from conventional motor threshold. Electrical stimulation of SC neurons biased choices in a manner predicted by the difference between the value representation and the value threshold. These results reveal a neural mechanism directly transforming absolute values to categorical choices within SC, supporting highly efficient value-based decision making critical for real-world economic behaviors.

## Abstract (web summary)

Value-based decision making involves choosing from multiple options with different values. The authors identify a neural mechanism that directly transforms absolute values to categorical choices within the superior colliculus and which supports value-based decision making critical for real-world economic behaviours.

## Introduction

During real-world economic behaviors, humans and other animals need to make efficient choices from multiple options based on their values. Economic and psychology theories propose that value-based decisions are made based on utility—a common scale of desirability^1,2^. To achieve transitivity of choices, the utility of one option should not depend on other options offered in the choice set^3^. Consistent with these theories, both imaging and electrophysiology studies found these utility-like representations—absolute value—were computed and represented in the ventromedial prefrontal cortex (vmPFC), the orbitofrontal cortex (OFC), and striatum^4–7^. However, how the brain makes categorical choices based on absolute values remains unclear.

Previous studies in sensorimotor regions, such as lateral intraparietal cortex (LIP), supplementary eye fields (SEF), and premotor cortex, mainly found neural correlates of normalized relative values, depending on the values of concurrently available options^8–11^. It is still unclear whether sensorimotor regions also encode absolute values and therefore be able to directly convert value information to choices.

To investigate this, we developed a multi-value saccade foraging task, inspired by the optimal foraging theory from behavioral ecology^12^. Monkeys were required to use saccadic eye movements to sequentially harvest rewards from an array of valued targets. Multiple targets shared the same values, so we could dissociate value coding of an option from whether it was chosen. The menu of available options dynamically changed as monkeys systematically harvested items from highest to lowest values. This allowed us to test whether value coding was dependent on the menu of available items.

We focused on the midbrain superior colliculus (SC), a key brain region in sensorimotor decisions. SC receives input from diverse sensory, motor, and reward related regions^13–18^, and sends output to saccade generating areas in brainstem^19,20^, and has been shown to play important roles in many cognitive processes such as target selection, attention and decision making^21–29^. We found that SC neurons encode both absolute value and context-dependent value threshold, suggesting a mechanism that could directly convert absolute values to categorical choice. We tested this possibility using electrical stimulation. We found that monkeys’ choice was biased in a manner predicted by the difference between absolute value representations and the context-dependent value threshold activity, providing a causal support for a thresholding mechanism of value-based decision making in SC.

## Results

**Fig. 1:**
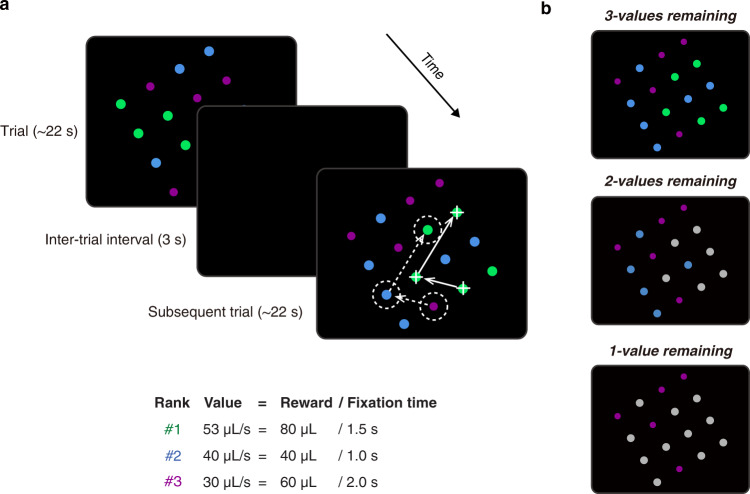
Saccade foraging task. **a** Two example trials of the saccade foraging task. The 4 × 4 array was composed of targets of three colors. Each target color was associated with a particular value determined by its water reward magnitude divided by the fixation time required to harvest this reward. When monkeys fixated a target for the pre-specified time, the color would turn into an equiluminant gray and corresponding reward was delivered, cueing the move to the subsequent target. In this example block, the rank of target values descended from green to blue to red. For illustration purposes, purple is used to represent red color in task paradigm. In successive trials, the association between color and value remained constant but the location of the colored targets within the array was randomized. The array size and orientation were tailored such that when the monkey was fixating a target (white cross), an adjacent target was positioned in the center of the pre-mapped response field (RF) of the isolated SC neuron (white dashed circle). The white arrows illustrate how the fovea and RF move in tandem as monkeys foraged targets in the array (More detail in Supplementary Movie 1). In a small number of experiments, larger response field eccentricities necessitated smaller 3 × 4 or 3 × 3 target arrays to fit on the visual display. **b** Examples of different menus. As monkeys tended to harvest targets in descending order of their value, the menu of items went from 3-values remaining (top), to 2-values remaining (middle), and finally 1-value remaining (bottom).

Two male monkeys (*Macaca mulatta*) were trained to perform the well-established delayed saccade task followed by a saccade foraging task, which developed from the optimal foraging theory (OFT)^12^ (Fig. 1). Anecdotally, there appears to be something particularly naturalistic and/or intuitive about this task as monkeys learn to efficiently forage in a single training session unlike many decision tasks that can take months or even years to learn. On each trial, monkeys were presented with a rectangular array composed of visual targets of 3 equiluminant colors. Monkeys harvested reward by fixating on a visual target for a pre-specified period of time. During each block, the fixation times required to harvest the water reward associated with each color were preselected from Supplementary Table 1. Consequently, all targets of the same color were associated with a particular target value [value = reward magnitude/fixation time]. Monkeys were free to fixate the targets in any order they chose. Once a target was harvested of its reward, it turned into an equiluminant gray color (Supplementary Movie 1). Between trials, the stimulus array disappeared for 3 s. When the array reappeared, the locations of the colored targets were shuffled but their value-color association remained intact.

### Monkeys were efficient value-based foragers

**Fig. 2:**
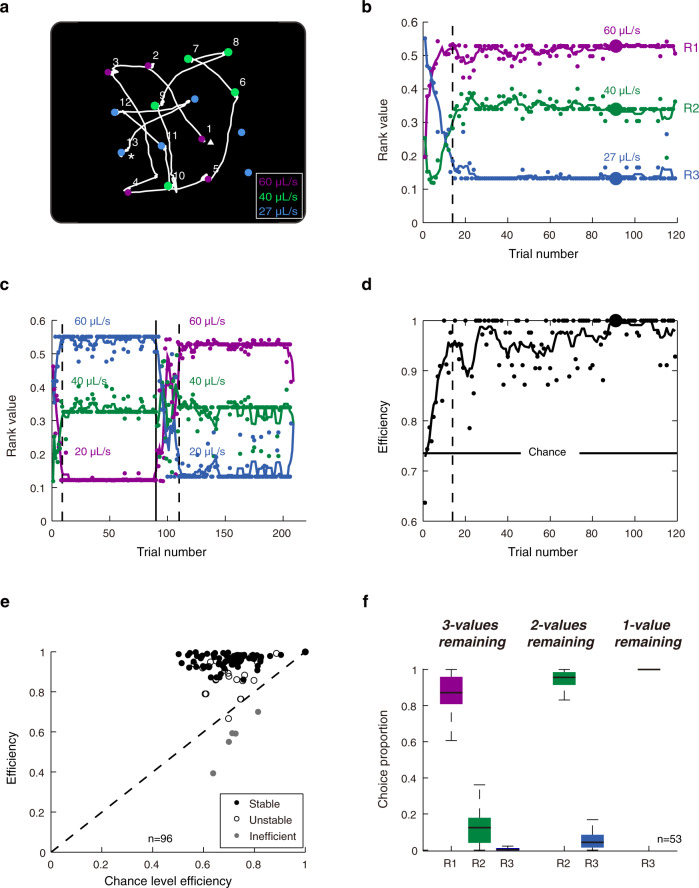
Monkeys were efficient foragers choosing targets in descending order of their value. **a** Scan path of the 91st trial in a representative experiment. This trial is shown in Supplementary Movie 1 along with audio of a simultaneously recorded SC neuron. The white line represents the eye trace and the numbers indicate the order of successfully harvested targets. The start and end of the trial is denoted with triangle and asterisk, respectively. In some instances, such as the fixation between eye position 12 and 13, the monkey did not hold fixation long enough to successfully harvest the target. These instances were not included in subsequent analyses. The colored numbers in the legend correspond with the value of the associated target colors. This particular trial/experiment is denoted by larger data points in subsequent panels. For illustration purposes, purple is used to represent red color in task paradigm. **b** Calculating the rank value of target colors. Each dot represents the value based on the order in which a particular class of colored targets was selected within a given trial. The colored lines represent the sliding average of rank value over five trials. The dashed line represents the time to behavioral acquisition (see “Methods”) when a stable value ranking was established as determined. The right colored numbers indicate the value ranking of each color which is measured from the order of median rank value across the block. **c** Same format as panel **b** except an unsignaled change in the target color-value relationship occurred at the solid line. Only the trials before the rule changes were included in population analyses. **d** The monkey’s efficiency at harvesting water for the representative experiment shown in (**b**). The black line represents the sliding average of efficiency over five trials. The horizontal line represents 95% confidence interval of chance efficiency by simulating random selection for 5000 trials. **e** Foraging efficiencies across all blocks plotted against their corresponding chance efficiencies. Only experiments that displayed significantly efficient and stable preference (black filled dots) were included in further analyses whereas inefficient (gray filled dots) and unstable blocks (unfilled black dots) were excluded. **f** Choice preferences of blocks in population neuronal analyses as menu transitioned from 3-values to 2-values, and to 1-value targets remaining. Source data are provided as a Source data file. For the boxplots, on each box, the central mark is the median, the edges of the box are the 25th and 75th percentiles, and the whiskers extend to the most extreme data points that the algorithm considers not to be outliers. Outliers are data points that are larger than Q3 + 1.5 × (Q3 − Q1) or smaller than Q1 – 1.5 × (Q3 − Q1), where Q1 and Q3 are the 25th and 75th percentiles, respectively (N.B.: The association between target color and value ranking was randomized between each experiment. However, for display purposes, purple, green, and blue will indicate the number 1, 2, and 3 value rankings, respectively, throughout the remainder of the paper.).

A critical aspect of the saccade foraging task was the monkeys were choosing under time pressure; that is, the target array disappeared before all targets could be harvested. On average, subjects were able to harvest 79 ± 6% targets. According to OFT^12^, when faced with such abundant prey items and time pressure, foragers should preferentially choose the highest valued available targets. Indeed, monkeys tended to choose scan-paths through the array in order of descending value (Fig. 2a; Supplementary Movie 1). At the beginning of a block, monkeys usually did not choose according to the OFT because they had not yet learned the association between target color and value (Fig. 2b; before dashed line). Learning tended to occur gradually over trials. But once the association between value and target color was established (i.e., ‘time to behavioral acquisition’—Fig. 2b; after dashed line), it tended to remain stable for the remainder of the block. This pattern of behavior was not simply due to an idiosyncratic preference for a particular color because choice preference changed as the association between color and value varied between experimental sessions or, occasionally without warning, within an experimental session (Fig. 2c).

Monkeys’ preferences, as quantified by rank value (see “Methods”), were influenced by the value of the colored targets. Most simply, the rank value of each color increased with its objective value (Supplementary Fig. 1a). Moreover, with wider ranges of objective values, monkeys’ preferences also became more distinct (Supplementary Fig. 1b) and less variable (Supplementary Fig. 1c) and, correspondingly, narrower ranges of objective values resulted in unstable and more variable preferences.

As monkeys learned to choose targets in descending order of value, their efficiency at harvesting water also increased (Fig. 2d). We defined the efficiency as the value of each chosen target divided by the highest value that was available within the array at that time. Across experimental sessions, monkeys’ efficiency after behavioral acquisition was better than chance (Fig. 2e; Wilcoxon signed-rank test, *P* = 2.3 × 10^−15^). Once behavioral acquisition was reached, the value rankings tended to remain stable thereafter (Fig. 2e—filled data points). The sessions with unstable value preferences (Fig. 2e—unfilled dots, see “Methods”) and the five sessions during which efficiency was significantly lower than chance (Fig. 2e—gray dots) were excluded from further analyses. Average efficiency of the remaining sessions was 0.96 ± 0.03. All subsequent analyses were performed only on those trials after time to behavioral acquisition was reached (see “Methods”).

Once a certain class of prey items was fully harvested, monkey’s choice behavior changed accordingly. Monkeys preferentially chose the highest value targets for a given menu (Fig. 2f). After the most valuable targets were exhausted (Fig. 2f; purple), monkeys chose the second most valuable targets (Fig. 2f; green), and after those were exhausted, they chose the least valuable targets (Fig. 2f; blue).

### The SC encodes both value and choice

We recorded 96 single neurons in the intermediate and deeper layers of the SC from two monkeys during the saccade foraging task. Sixty-five experiments satisfied our behavioral criteria (see “Methods” and Fig. 2e), and of those, 53 neurons were considered task modulated (i.e., displayed significant delay-period activity and had well-defined response fields) and were included in the following analyses.

**Fig. 3:**
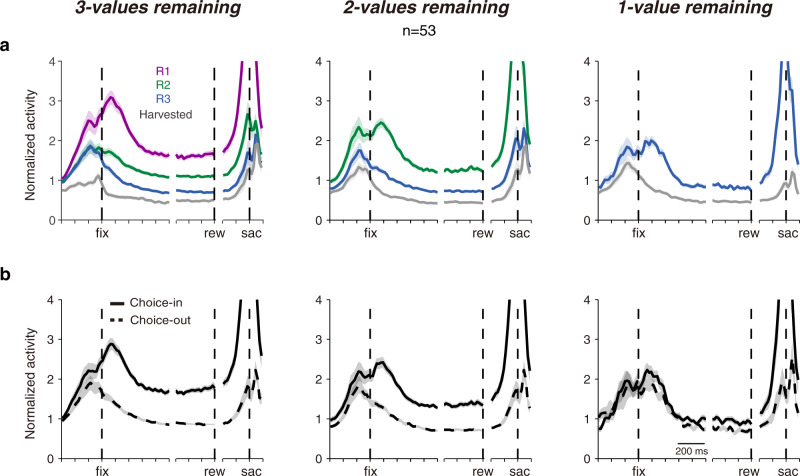
Population neuronal activity reflected target value in the response field and predicted upcoming saccade choice. Note that plots represent **a** the value of the target in the RF (line color—R1 (purple) denotes highest rank value; R2 (green) denotes middle rank value; R3 (blue) denotes lowest rank value; harvested (gray) denotes previously harvested target with no value), or **b** choices directed into (Choice-in) or out (Choice-out) of the response field rather than properties of the currently fixated target. The left side of each panel is aligned on the beginning of fixation (fix), the middle is aligned on reward delivery (rew), and the right is aligned on saccade onset (sac). The total duration of the spike density waveforms could not be shown because the fixation time varied across targets and experiments. The shaded regions surrounding each line represent SEM.

SC activities were correlated with value ranking of targets in the neuronal response fields (RF; Fig. 3a). To be clear, the order in which the foveae harvested targets provided us with a behavioral measure of the subjective value ranking of the colored targets (Fig. 2), however, each recorded SC neuron analyzed a target adjacent to the current foveae location and in its RF (see Fig. 1a and gray shading in Supplementary Movie 1). Whereas the foveae harvested targets in a fairly strict descending order of their value, the targets that entered the neuron’s RF were largely random. Within each menu, neuronal activity was scaled by the value of the target in the RF (Fig. 3a). Moreover, if a previously harvested gray-colored target entered the RF, this elicited the least SC activity. The latter may represent the baseline sensory activation because these harvested targets had no value and were virtually never the target for a saccade. However, neuronal activity was not only modulated by value in the RF, but was also highly predictive of choice. Neuronal activity leading up to choices directed toward the RF target (Fig. 3b; Choice-in) was significantly stronger than activity preceding a choice to targets outside the RF (Fig. 3b; Choice-out).

This analysis is difficult to interpret because monkeys are more likely to choose high-valued targets (see Fig. 2f); thus, value and choice encoding is difficult to disentangle. An advantage of our saccade foraging task is that there are many potential targets that share the same value but are not necessarily the target of the next choice, which allows us to dissociate the contribution of each to neuronal activity.

**Fig. 4:**
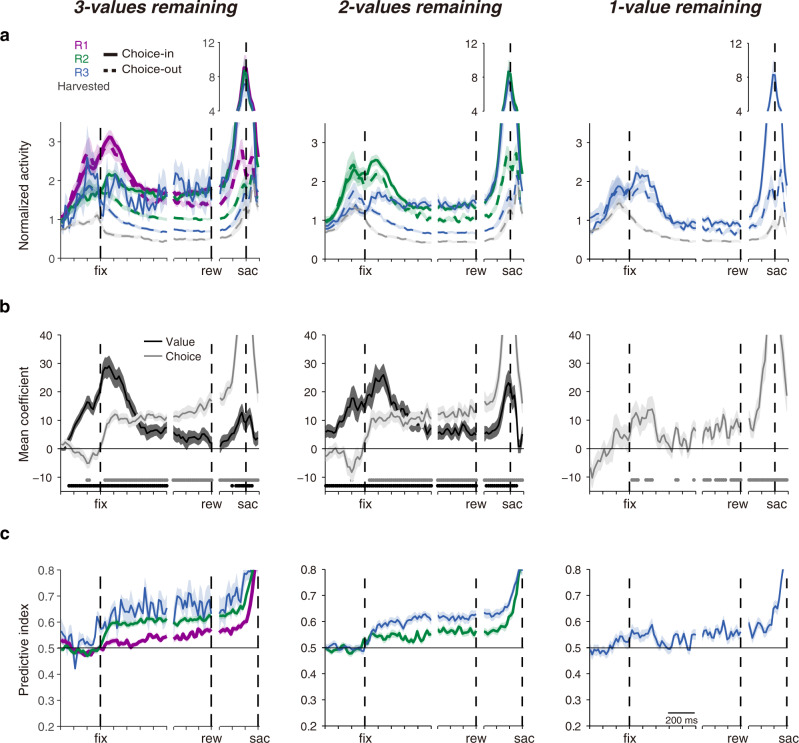
The evolution of value and choice signals in SC activity across 3 menus. **a** Normalized neuronal activity was segregated based on the rank value of the target in the response field and whether a saccade was ultimately directed to the response field target (solid lines) or a target outside the response field (dashed lines). Otherwise, the same format as Fig. 3. **b** The evolution of the population regression coefficients for value ranking (black) and choice direction (gray). There is no value plot in the rightmost 1-value remaining panel because all remaining targets had the same, lowest value. The time points with statistically significant regression coefficients by value ranking (black dots) and choice direction (gray dots) was shown at the bottom of the panel (*t* test, *P* < 0.05, with false-discovery rate correction). **c** The evolution of neuronal choice selectivity toward targets of different value rankings. The receiver-operating characteristic analyses were done between activities associated with choices toward the response field target versus targets outside the response field that shared the same value. All shaded regions represent SEM.

After the variables were isolated from each other, SC activity still remained correlated with both target value in, and upcoming choice toward, the RF (Fig. 4a); but each displayed a different time course. Regression analyses showed the value signal began to significantly increase ~300 ms before the target was even fixated (i.e., Fig. 4b; horizontal black lines) and peaked within 200 ms of a new target entering the RF. Perhaps this pre-fixation activity represented predictive ‘remapping’ of value signals akin to the sensory remapping that occurs when saccades will bring visual stimuli into SC response fields^30,31^. In contrast, the choice signal increased significantly only after the fixation period began (Fig. 4b; horizontal gray lines). Overall, there was a ‘value-to-choice’ transformation such that value signals were dominant early during a new fixation and choice signals became dominant as the fixation period progressed (Fig. 4b). These results were highly consistent across both monkey subjects (Supplementary Figs. 2 and 3). These observations were consistent with previous studies trying to dissociate the value coding and choice process using two-alternative forced-choice (2AFC) task paradigm^32–34^, however, our results show important mechanistic details about this transformation that will be described below.

### SC neurons represent value threshold and value of targets during late fixation

Slightly before and during fixation of a new target, neuronal activity primarily reflected the value ranking of the target in its RF. Immediately upon establishing fixation, however, the choice signal quickly began to ramp up (Fig. 4b). As the fixation period progressed, there was a marked differentiation in SC activity based on whether the monkey would ultimately look to the RF target or elsewhere.

**Fig. 5:**
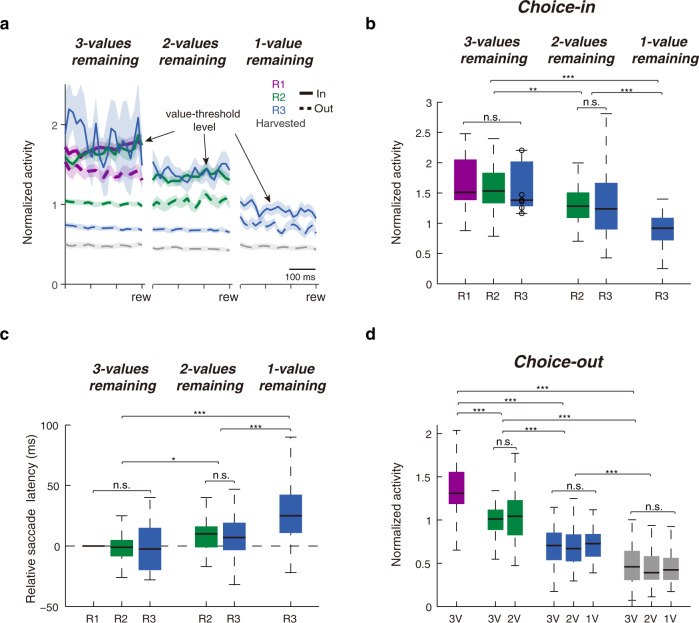
Menu updating of the value-threshold level and value representations. **a** Normalized neuronal activity during the late fixation period (last 300 ms before reward delivery, rew). Same format as Fig. 4a. **b** Neuronal activity associated with choice-in conditions. For conditions from left to right *n* = 53, 51, 7, 53, 44, and 47 blocks. For multiple-menu comparisons (labeled with any number of *) from left to right *P* = 2.6 × 10^−3^, 5.8 ×  10^−8^, 7.7 × 10^−5^. **c** Relative saccade latencies toward different value ranking targets across the 3 menus. All latencies are calculated relative to the 1st value ranking targets in the 3-values remaining menu. For conditions from left to right *n* = 53, 50, 14, 53, 40, and 53 blocks. For multiple-menu comparisons (labeled with any number of *) from top to down *P* = 1.7 × 10^−10^, 3.4 × 10^−7^, 0.011. **d** Neuronal activity associated with choice-out conditions. For conditions from left to right *n* = 49, 53, 49, 53, 53, 39, 53, 53, and 53 blocks. For multiple-value comparisons (labeled with any number of *) from top to down *P* = 8.5 × 10^−66^, 3.3 × 10^−42^, 4.0 × 10^−14^, 1.1 × 10^−46^, 1.4 × 10^−18^, 4.9 × 10^−14^. Data are presented for each value ranking when there were three values (3 V), two values (2 V), or one value (1 V) targets remaining in the array. *n* in (**b**–**d**) represents the number of blocks with more than 3 cases for the condition. For (**b**–**d**), source data are provided as a Source data file. For the boxplots, on each box, the central mark is the median, the edges of the box are the 25th and 75th percentiles, and the whiskers extend to the most extreme data points that the algorithm considers not to be outliers. Outliers are data points that are larger than Q3 + 1.5 × (Q3 − Q1) or smaller than Q1 − 1.5 × (Q3 − Q1), where Q1 and Q3 are the 25th and 75th percentiles, respectively (n.s., nonsignificant, *P < 0.05, **P < 0.01, ***P < 0.001; n-way ANOVA tests, post hoc tests were done with Bonferroni correction).

A stable, common level of activity was reached in the SC if the monkey would ultimately choose the RF target regardless of its value (Fig. 4a; clearer in Fig. 5a—solid lines). Within each given menu, the choice-in neuronal activity associated with each value ranking reached a common level during the late period of fixation (Fig. 5b; n-way ANOVA test, effect of value, F_2, 250_ = 0.31, *P* = 0.73; see Supplementary Fig. 4a, b for individual monkey data). This common choice-in level of activity might reflect value threshold, which represents the selected option in value-based decision making. Consistent with this proposal, both regression (Fig. 4b) and signal detection (Fig. 4c) analyses showed that choice signals built up and reached a plateau shortly after a new target was fixated. Moreover, behavioral results showed that, within a given menu, choice-in saccade latencies were the same regardless of the value of the saccade targets (Fig. 5c; n-way ANOVA test, effect of value, F_2, 258_ = 0.01, *P* = 0.99; see Supplementary Fig. 4c, d for individual monkey data). This pattern of reaction times was consistent with a neural signal that starts from a common value-threshold level that ramp up to a fixed saccade threshold.

Conversely, if the RF target was not selected for the subsequent saccade, SC activity remained strongly correlated with its value (Fig. 5a—dashed lines; Fig. 5d; n-way ANOVA test, effect of value, F_3, 449_ = 174.93, *P* = 4.1 × 10^−75^; see Supplementary Fig. 4e, f for individual monkey data).

### Menu updating of the value-threshold level

To understand how choice mechanisms accommodate menu changes, we compared SC activity across menus (Fig. 5a). Although the value-threshold level remained constant within a menu regardless of the value of the chosen target, it systematically decreased across menus (Fig. 5b; n-way ANOVA test, effect of menu, F_2, 250_ = 16.86, *P* = 1.4 × 10^−7^; see Supplementary Fig. 4a, b for individual monkey data). This decreasing value-threshold level was not the result of harvesting individual targets but occurred during the transition between menus after entire value classes were harvested (Supplementary Figs. 5c and 6). Nor did the value of the currently fixated target have an effect on value-threshold level across menus (Supplementary Fig. 7). These menu-dependent changes in the value-threshold level were reflected in the saccade latency (Fig. 5c; n-way ANOVA test, effect of menu, F_2, 258_ = 23.8, *P* = 3.3 × 10^−10^; see Supplementary Fig. 4c, d for individual monkey data). As the value-threshold level decreased with fewer menu items, it presumably got further from the hard saccade threshold in the SC^19,20^ resulting in saccade latencies that increased as the menu items decreased from three to two to one. Together, this suggests that the value-threshold level is dynamically updated across the SC map with changes in menu items.

### SC value representations during late fixation are invariant with menu

If neuronal activity was segregated only by the value of the target in the RF, it provides a somewhat misleading picture. Activity associated with each value ranking increased with fewer menu items during both the early (300 ms after fixation beginning; Supplementary Fig. 8a) and late period of fixation (300 ms before the reward delivery; Supplementary Fig. 8b). This was consistent with previous studies showing sensorimotor regions represent menu-variant value^9,10^.

However, when neuronal activity was segregated by both value and choice across menus a different picture emerges. During late fixation, the choice-out activity of each value ranking remained stable regardless of number of array items (Supplementary Fig. 5d) or across menus (Fig. 5d; n-way ANOVA test, effect of menu, F_2, 449_ = 0.27, *P* = 0.76); that is, value representation during late fixation was menu-invariant. In contrast, the early visual activity immediately after fixating a new item was menu-variant with respect to value for both choice-in (Supplementary Fig. 9a) and choice-out conditions (Supplementary Fig. 9b). This early visual response varied across menus and not simply as the number of available array items decreased (Supplementary Fig. 5a, b). Indeed, most previous studies showing a menu-variant value representation^9,10^ have focused on a comparable early period of visual processing.

Last, we tackle the question of whether the menu-invariant representation of value observed during late fixation (Fig. 5a, d) reflected an ordinal ranking of value or a more graded measure of subjective value. By only focusing our analyses under conditions with highly stable preferences (Fig. 2e, filled circles), it is difficult to get a graded measure of values to test this question. Therefore, here we also included those experiments in which the monkey’s preferences were less stable over a block of trials (Fig. 2e, unfilled circles) and, presumably, the subjective values were not as distinct as stable preference blocks. We observed a statistically significant correlation between the rank values of targets across experiments and neuronal activity during late fixation (Supplementary Fig. 10), suggesting that the SC value coding did not reflect simple ordinal value rank but subjective value.

### Balance between value-threshold level and value representations

**Fig. 6:**
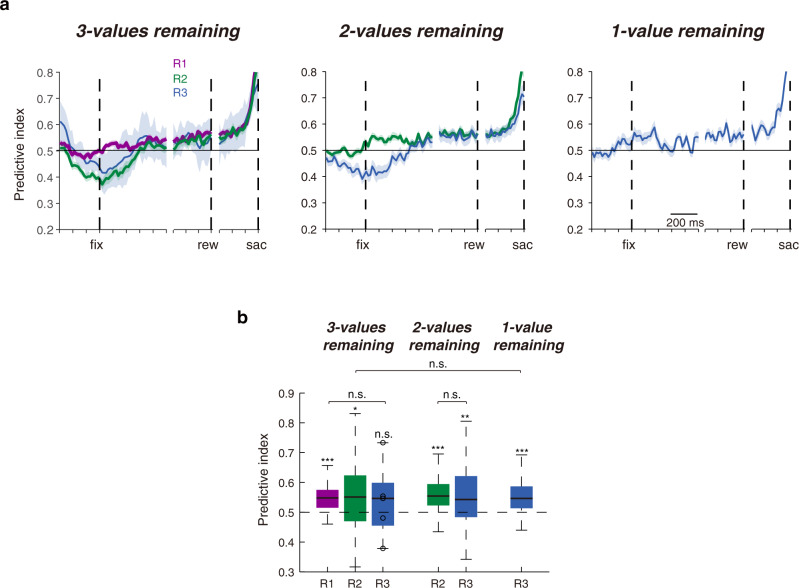
Neuronal selectivity remained remarkably constant across conditions and menus. **a** The evolution of neuronal choice selectivity as determined by receiver-operating characteristic analysis when comparing choice-in activities of each value ranking with the highest available choice-out activity. All shaded regions represent SEM. **b** The average predictive indexes during the late fixation period from (**a**). For conditions from left to right *n* = 49, 48, 5, 48, 38, and 33. *n* represents the number of blocks with at least five cases for the condition. Data are presented for each value ranking when there were three values, two values, or one value ranking targets remaining in the array. For each value ranking, two-sided, one-way *t* test, from left to right, *P* = 4.8 × 10^−8^, 0.040, 0.54, 7.2 × 10^−8^, 0.0053, and 3.6 × 10^−5^, respectively. N-way ANOVA tests, factor of value, *P* = 0.65, factor of menu, *P* = 0.36. Source data are provided as a Source data file. For the boxplots, on each box, the central mark is the median, the edges of the box are the 25th and 75th percentiles, and the whiskers extend to the most extreme data points that the algorithm considers not to be outliers. Outliers are data points that are larger than Q3 + 1.5 × (Q3 − Q1) or smaller than Q1 − 1.5 × (Q3 − Q1), where Q1 and Q3 are the 25th and 75th percentiles, respectively (n.s. nonsignificant, *P < 0.05, **P < 0.01, ***P < 0.001).

An upshot of having a menu-variant value-threshold level but menu-invariant value representations is that neuronal selectivity for choice remained remarkably consistent under all conditions. When we performed signal detection analyses for the choice-in activities of each value ranking versus the highest available choice-out value representations on the SC map, the neuronal selectivity remained approximately 55% during the steady-state late period of fixation (Fig. 6a). This 55% neuronal selectivity remained unchanged regardless of the value of the chosen target or the composition of the targets in the menu (Fig. 6b; n-way ANOVA test, effect of value, F_2, 216_ = 0.43, *P* = 0.65, effect of menu, F_2, 216_ = 1.03, *P* = 0.36). This result suggests that there may be an inherent equilibrium achieved between the value-threshold level and value representations.

### Manipulating SC activity biases choice in a manner predicted by SC recordings

As both the value-threshold level and value representations were found in SC, we propose a thresholding mechanism underlying such foraging choices in which an option is selected when its value representation exceeds the value-threshold level. To test this hypothesis, we applied electrical micro-stimulation in SC during the late period of fixation. More specifically, we hypothesize that the efficacy with which micro-stimulation biases choice is a function of the neuronal distance between the value representations at the stimulation site and the menu-dependent value-threshold level (i.e., the distance between the relevant dashed line and solid lines in Fig. 5a).

Our goal was to bias selection processes on the SC map without directly triggering a saccade itself, so we used sub-threshold electrical micro-stimulation to increase SC activity^23^. Two lines of evidence suggest that micro-stimulation was sub-threshold. First, we only included experiments if no saccades were triggered during the micro-stimulation period (Supplementary Fig. 11a; bottom panel gray box). Second, we did not observe any difference in saccade latency between stimulation and control conditions both in the example block (Supplementary Fig. 11b; Wilcoxon rank-sum test, *P* = 0.60) and across the population of stimulation sites (Supplementary Fig. 11c; Wilcoxon signed-rank test, Choice-in, *P* = 0.36; Choice-out, *P* = 0.74).

**Fig. 7:**
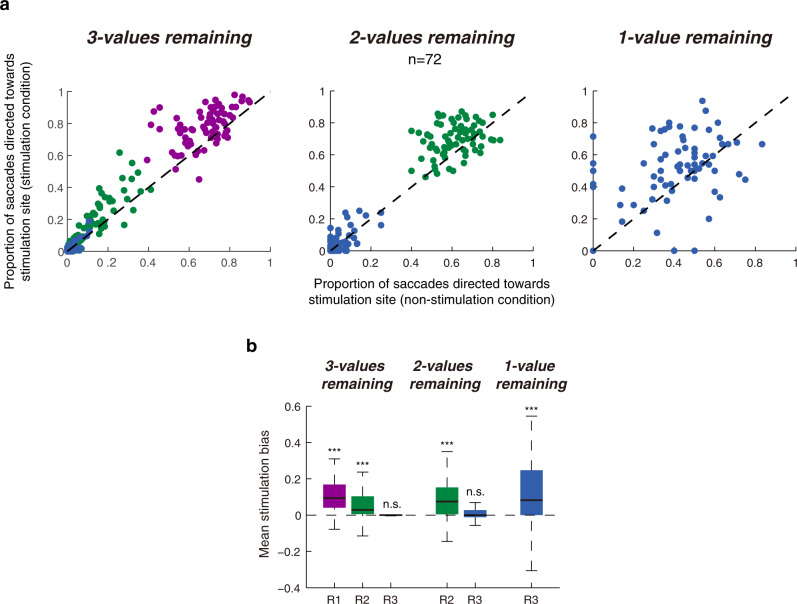
Sub-threshold micro-stimulation applied during the late fixation period biased choice. **a** The proportion of choices directed toward the stimulation site target in stimulation condition vs. non-stimulation control condition across the 3 menus. Purple, green, and blue denote when 1st, 2nd, and 3rd value ranking targets were located at the stimulation site, respectively. The dashed line represents the line of unity. **b** Difference in the proportion of saccades directed toward the stimulation site under stimulation condition minus non-stimulation condition. All the 72 blocks in stimulation experiments were included. Wilcoxon signed-rank test, two-sided, from left to right, *P* = 3.2 × 10^−10^, 8.7 × 10^−8^, 0.90, 1.2 × 10^−6^, 0.28, and 1.3 × 10^−4^, respectively. Source data are provided as a Source data file. For the boxplots, on each box, the central mark is the median, the edges of the box are the 25th and 75th percentiles, and the whiskers extend to the most extreme data points that the algorithm considers not to be outliers. Outliers are data points that are larger than Q3 + 1.5 × (Q3 – Q1) or smaller than Q1 – 1.5 × (Q3 – Q1), where Q1 and Q3 are the 25th and 75th percentiles, respectively.

Next, we assessed whether this sub-threshold, micro-stimulation could affect choice as our predictions. The proportion of choices directed toward the stimulation site increased significantly after stimulation compared to the non-stimulation control condition (two-sided, paired *t*-test, *t*(71) = 8.57, *P* = 1.5 × 10^−12^). However, micro-stimulation did not bias choices toward all targets equally. Micro-stimulation biased choices predominantly when there were high-value targets at the stimulation site (Fig. 7). When there were three values in the menu, micro-stimulation significantly biased choices toward highest valued targets (mean = 0.104 ± 0.013), less so to middle-ranking targets (mean = 0.053 ± 0.009), and not at all to lowest valued targets (mean = 0.000 ± 0.002). Once the highest valued targets were all harvested, the 2nd ranked targets became the most valuable, and micro-stimulation exerted a stronger biasing effect toward them than when there were 3 menu items remaining (mean = 0.083 ± 0.014). Only when there was 1-value remaining in the menu did micro-stimulation exert a significant biasing effect toward the lowest valued targets at the stimulation site (mean = 0.105 ± 0.027). Therefore, the overall biasing effect of micro-stimulation on choice was both value- and menu-dependent (Fig. 7b; n-way ANOVA; effect of value, F_2, 427_ = 19.6, *P* = 7.2 × 10^−9^; effect of menu, F_2, 427_ = 17.2, *P* = 6.7 × 10^−8^) and closely mirrored the value- and menu-dependent patterns of behavioral choice (Fig. 2f) and SC activities (Fig. 5a). Consistent results were observed in both monkey subjects (Supplementary Fig. 12) and in both similar-value and different-value blocks (Supplementary Fig. 13a). The biasing effect depended on the rank value difference between valued targets; stronger biasing effect on the lower valued targets was observed when the rank value of the low-value targets was closer to the high-value targets (Supplementary Fig. 13b-d).

## Discussion

**Fig. 8:**
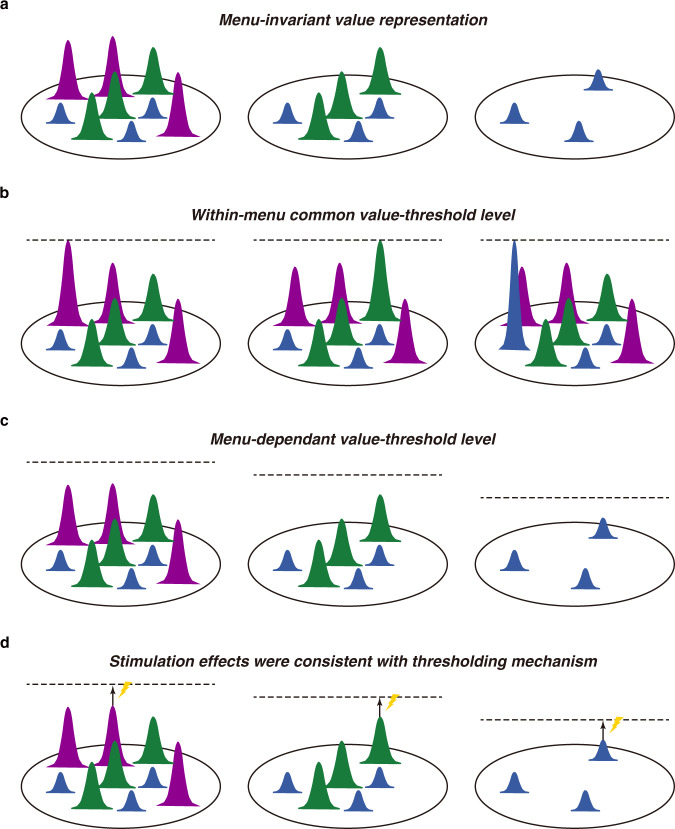
Four findings in the SC support for thresholding mechanisms in value-based decision making. The Gaussian curves represent late fixation population activities on the SC map associated with the highest (purple), middle (green), and lowest (blue) valued targets in the visual array. The dashed line represents the value-threshold level. **a** The value of targets was represented across the SC in an absolute manner that did not vary as the menu decreased from 3-values remaining (left) to 2-values remaining (middle) to 1-value remaining (right). **b** Within a given menu, neuronal activity associated with the selected option reached the same value-threshold level regardless of whether the highest (left), middle (middle), or lowest (right) valued target was chosen. **c** The value-threshold levels systematically decreased as the menu changed from 3-values remaining (left), 2-values remaining (middle), and 1-value remaining (right). **d** Stimulation effect was a function of the distance between absolute value representations and value-threshold levels.

We employed a foraging task to examine the role of the primate SC in value-based saccade decisions. Multiple targets sharing the same value and the numerous successive choices afforded by this task allowed us to examine neural processing both when value was dissociated from choice and across changes in the menu of value items. Monkeys performed this task at a near optimal level as predicted by optimal foraging theory (Fig. 2). At the neuronal level, we observed four findings as illustrated in the schematic model in Fig. 8. First, value was represented in an absolute or menu-invariant manner across the SC map during late period of fixation (Fig. 8a). Second, SC neurons also represented value-threshold levels that predicted the upcoming choice (Fig. 8b). Third, these value-threshold levels were menu-dependent and decreased as classes of menu items were removed from the choice array (Fig. 8c). Fourth, electrical stimulation of SC neurons biased choice in a manner predicted by the difference between absolute value representations and value-threshold levels (Fig. 8d). These findings provide direct evidence for a thresholding mechanism for transforming absolute values to a motor choice; that is, a particular saccadic action is selected once the value representation exceeds the menu-dependent value-threshold level in SC.

### Absolute value representation in SC

It has been proposed that relative, rather than absolute, value is encoded in sensorimotor regions^35,36^. Surprisingly, we found absolute value representation in SC, providing direct evidence for absolute value representation in sensorimotor regions. One possible explanation is that we focused on a later time period in our task compared with previous studies. When we examined the time period immediately after a new stimulus entered the response field, we found relative or menu-dependent value representations similar to those observed in other sensorimotor regions^9–11^ (Supplementary Fig. 9). However, activities during this initial processing period may be strongly modulated by saliency or attention^27,37,38^. Another possible explanation is that many previous studies employed a block task design^8^ that may have allowed slow adaptation of neuronal representation across many trials^39^. A third possible explanation is that the value coding was associated with motor preparation in some previous studies. As shown in Supplementary Fig. 8, when separating the neuronal activities only by value, even during the late period of fixation, the SC neuronal activity of one value appeared to be dependent on menus. Finally, the absolute coding observed in our study could also be attributed to the multi-value and dynamic nature of our foraging task, where encoding absolute value of different options may be more efficient than encoding relative value of them in each menu and updating that across menus.

Where did the absolute value signal in SC come from? The SC receives diverse input from many cortical and subcortical areas, including the dorsolateral prefrontal cortex (dlPFC), ventral premotor area, frontal eye field (FEF), LIP, and basal ganglia, many of which are related to value representation^25,35,40^. Because the striatum was reported to encode absolute value of actions^7^, basal ganglia input could be a source of the absolute value signal in the SC. Future studies are needed to investigate whether cortical regions with direct projections to SC also encode absolute value during the late fixation. Pathway-specific perturbation experiments could provide further insights into which upstream regions contribute to the absolute value coding in the SC.

### SC neurons encode value threshold

We found threshold-like activity in the SC during the late period of fixation. In the context of perceptual decision making, it has been proposed that SC neurons could passively detect the crossing of decision threshold that was determined by basal ganglia^41^. Here, we observed SC activity directly representing a decision threshold in the context of value-based decision making, and this threshold is modulated by menu. This menu-dependent value threshold is consistent with ideas that decision threshold could be adjusted under conditions such as urgency or confidence estimation in previous studies of perceptual decisions^42,43^. A recent study showed the difference between choice-in and choice-out activity in the SC corresponds to decision criteria of perceptual decisions^24^. Similar to our SC recordings, their results could also be explained by context-dependent decision threshold, where changes of choice-in activities in different contexts led to changes in decision criteria.

Another question is how the value threshold was computed. Our results show that, despite the decrease in threshold-like activity across menus, there was a consistent balance between the threshold-like activity and value representations (Fig. 6). This balance could be achieved by local inhibition in the SC^44^ or the global inhibition by the projection from basal ganglia^40^. Theoretical and experiment studies suggested that the perceptual decision threshold was determined by the strength of cortico-striatal synapses^41,42^, which may also play an important role in computing the value threshold in the SC.

### Thresholding mechanism for value-based decision making

Our data support a thresholding mechanism for value-based decision making. Previous studies showed that sensorimotor regions primarily encode relative value for action selection, consistent with the winner-take-all model^35,36,45^ or drift-diffusion model^46,47^. The thresholding mechanism found in our study could convert absolute values directly to motor choices by comparing with a prescribed value threshold. In the meanwhile, absolute value coding enables stable representation of preferences, while menu-dependent value threshold enables context-dependent flexible choices. Together, our findings unravel an efficient and flexible neural mechanism underlying value-based decision making.

## Methods

### Animal preparation

Two male rhesus monkeys (*Macaca mulatta*; D, 12 kg, and R, 8 kg) participated in this study. Animals were under the close supervision of the Institute veterinarian. All procedures were approved by the Animal Care and Use Committee of the Institute of Neuroscience, Chinese Academy of Sciences, Shanghai, China.

Throughout the experiments, monkeys were seated in a primate chair with their heads firmly fixed via the head-post in the implant. The monkeys faced a LED screen (60 Hz refresh rate) 60 cm away which spanned ±30° vertical and ±45° horizontal of the central visual field. The position of the left eye was sampled at 500 Hz by an EYELINK1000 infrared eye tracker (SR Research). Behavioral tasks were under the control of Monkeylogic software (http://www.monkeylogic.net/). All data analyses were performed offline using custom built code in MATLAB (version R2014a, Mathworks Inc).

### General procedures

Both single-neuron recording and sub-threshold micro-stimulation were performed in the intermediate and deeper layer of the SC (between 1.0 and 3.0 mm below, and tangential to, the surface of the SC).

Tungsten microelectrodes (FHC Inc.) were lowered with a Microdrive (NAN Instruments) through 23 gauge, 42-mm long stainless steel guide tubes (with 10-mm spacer) attached to a Crist grid (Crist Instruments Co., Inc.). Single-cell discharges were sampled at 22 kHz using the AlphaLab SnR System and subsequently offline sorted using Spike 2 (version 7.15; CED, Cambridge Electronic Design Limited).

### Behavioral tasks

Monkeys performed two behavioral tasks. The delayed saccade task was used to characterize neuronal properties and map the center of their response fields (RF). The saccade foraging task was used to correlate neuronal activities to value and choice. Separately, we applied stimulation during the saccade foraging task to determine how perturbing SC activity would alter foraging choices.

#### Delayed saccade task and response field identification

The delayed saccade task was performed before the saccade foraging task in both neuronal recording and micro-stimulation blocks^48^. Trials started when a fixation point appeared at the center of the screen. After monkeys acquired and fixated the fixation point for 1000 ms, a single target point appeared in the periphery. The monkeys were required to maintain fixation at the center point for a random delay period (600–800 ms). At the end of the delay period, the fixation point disappeared, cueing the monkey to initiate a saccade toward the peripheral target. Monkeys received a liquid reward if they initiated a saccade within 1000 ms and maintained fixation within 3 degrees of the target and held it for 300 ms. Both the fixation point and target stimulus shared the same luminance and size (4.20 cd/m^2^ luminance, 0.5° visual radius) as targets in the saccade foraging task.

The center of an isolated single neuron’s (recording experiments) or multi-units’ (stimulation experiments) RF was defined as the location relative to central fixation that was associated with the most vigorous activity following target appearance and during target-directed saccade in the delayed saccade task. For stimulation experiments, we also used supra-threshold stimulation to verify that the stimulation-induced vector and recorded RF vector displayed close correspondence. Our experiments focused on SC sites with small saccade vectors ranging from 3 to 16° (6.4 ± 2.4° for 76 blocks of 16-target arrays; 12.9 ± 1.3° for 13 blocks of 12-target arrays; 14.0 ± 3.0° for 7 blocks of 9-target arrays). Larger vector sites would not allow for a sufficient number of targets in the array to also fit on the visual display during the saccade foraging task.

#### Saccade foraging task

In the saccade foraging task (Fig. 1), each trial began with the display of a grid array of circular stimuli on the screen in front of the subject. The grid was rotated, scaled and the number of targets adjusted (3 × 3, 4 × 3, or 4 × 4) such that when fixating a target, a nearby target would be located in the center of the neuron’s RF (except, of course, when fixating the outermost contralateral column). All visual targets were identical in terms of luminance and size (4.20 cd/m^2^ luminance, 0.5° visual radius), but could either be red, green, or blue. During each trial, target colors were represented in equal proportions, but their locations on the grid were randomly shuffled between trials. Throughout a block of trials, visual targets that shared the same color were associated with the same value as defined by reward magnitude divided by fixation time (value = reward magnitude/fixation time; see Supplementary Table 1). That is, monkeys harvested a specified volume of liquid reward associated with a colored target by fixating it for a particular duration. Once harvested, the target’s color turned into an equiluminant gray and could not be harvested again during that trial. Prior to each block, both reward magnitudes and fixation times were selected randomly with replacement while excluding identical combinations from Supplementary Table 1. Monkeys were free to harvest targets in any order they chose. However, we adjusted trial durations such that there was not enough time to harvest all targets. Trial duration was set manually by the experimenter according to the total fixation time of all the targets in the grid (about 1 or 2 s lesser). The trial duration was fixed during a given block. The average trial duration was 20.5 ± 4.5 s for monkey D and 19.6 ± 4.1 s for monkey R. On average, 79 ± 6% of the targets were harvested. In a few sessions, we changed the value-color association without warning (e.g., Fig. 2c). Trials after rule changes were never included in subsequent analyses and thus each neuron contributed only once to all population analyses.

In contrast to the recording experiments, only two value associations were used in the stimulation saccade foraging task: the similar-value condition and the different-value condition. Under the similar-value condition, reward magnitude (μL)/fixation time (s) associations were 30:1, 45:1.5, and 60:2, such that the three colors shared the same objective value of 30 μL/s. Under the different-value condition, reward magnitude (μL)/fixation time (s) associations were 20:2, 40:1.5, and 60:1, such that the values associated with the 3 colors were 10, 27, and 60 μL/s, respectively. The results from both value conditions were consistent therefore we analyzed them together.

### Behavioral analysis

#### Rank value

The rank value quantified the relative preference for each color using the order in which each target was chosen (e.g., Fig. 2b and c). Importantly, this measure does not provide an exact measure of the subjective value of each color but it does provide an ordinal rank value for each color. This measure is derived from the nonparametric Kruskal–Wallis test^49^. The first chosen target in a given trial was given a score of *N*, where *N* is the total number of targets initially presented in the grid. The next chosen target was given a score of *N* − 1 and scores decreased by 1 each time until the last choice:

1 $${S}_{i}=N-(i-1)$$

Where ${S}_{i}$ is the score of the *i*th chosen target.

All targets that were not selected by the end of the trial were given equal scores that was the mean of the scores that they jointly occupy:

2 $${S}_{i \,> \, n}=\frac{{\sum }_{j=1}^{N-n}\,j}{N-n}$$

Where *n* is the number of targets harvested, ${S}_{i \,> \, n}$ is the score of each target that was not selected by the end of the trial and *j* is each of the remaining scores.

The rank value (RV) is the summed score of that color ($\sum {S}_{C}$) normalized by dividing by the summed score of all colors. Because the number of targets cannot be equally divided amongst the 3 colors in the 16-target conditions, we adjusted the equation by dividing the summed score of each color ($\sum {S}_{C}$) by the number of targets of that color (*N*_*C*_).

3 $${{{{RV}}}}_{C}=\frac{\sum {S}_{C}/{N}_{C}}{\sum {S}_{R}/{N}_{R}+\sum {S}_{G}/{N}_{G}+\sum {S}_{B}/{N}_{B}}$$

Where *C* is one of the colors R, G, or B, and ${S}_{C}$ represents the score of each target that features the color *C*.

Sliding average of *RV*_*C*_ over five trials was used to reduce the trial-by-trial variability. The value ranking was ordered by the median *RV*_*C*_ in a block.

#### Time to behavioral acquisition

To quantify when the subjects learned the color-value associations, we did pairwise comparison between the rank values across the three colors within a moving 5-trial window with 1-trial steps (Kolmogorov–Smirnov test, one-sided). Time to behavioral acquisition was the trial when the *RV*_*C*_ was significantly separated in order of value ranking of the block and remained so for at least five consecutive trials (e.g., Fig. 2b–e).

#### Efficiency

We defined the efficiency, *E*_*i*_ of each choice (e.g., Fig. 2d and e) as the value associated with that choice, *V*_*i*_, divided by the highest available value present at the time that the choice was made, *V*_*Hi*_:

4 $${E}_{i}=\frac{{V}_{i}}{{V}_{{Hi}}}$$

The foraging efficiency associated with each trial was defined as the sum of the efficiencies of all the choices made in that trial, divided by the number of chosen targets or, in other words, the average efficiency of the trial:

5 $${E}_{{{\mathrm{trial}}}}=\frac{{\sum }_{i=1}^{n}{E}_{i}}{n}$$

Where ${E}_{{{\mathrm{trial}}}}$ denotes the foraging efficiency of the trial, ${E}_{i}$ denotes the efficiency of the *i*th choice, and *n* denotes the number of choices made in the trial.

To examine if subjects’ efficiency was significantly higher than random choices, for each block the chance efficiency was computed by simulating a random selection process over 5000 iterations. Only blocks with significantly higher efficiency than chance efficiency were included in subsequent analyses. Trials before behavioral acquisition were not included in the population efficiency calculation.

#### Exponential fit

For Supplementary Fig. 1, we fit (*a*, *b*) data with the following exponential equations:

6 $$y=a\left({1-e}^{-{bx}}+c\right)$$

(*c*) data with exponential equations:

7 $$y=a\left({e}^{-{bx}+c}\right)$$

#### Behavioral criteria

Datasets had to satisfy three behavioral criteria to be included in further analyses.

1. Only trials after behavioral acquisition were analyzed.
2. Only trials in which both the order of single-trial *RV*_*C*_ and sliding *RV*_*C*_ were consistent with the overall value ranking of the block were included in subsequent analyses. However, entire blocks were removed from analysis if there were more than 25% inconsistent trials within a block (i.e., unstable blocks). One exception to this rule occurred during some similar-value, stimulation blocks when preferences occasionally switched for extended periods (at least 10 trials). Analyses followed these extended preferences rather than average value preferences across the block.
3. Last, total useable trials within a block must exceed 50 for recording blocks and 100 for stimulation blocks.

Based on these criteria, 70 of the 96 blocks in recording experiments met the behavioral criteria and 72 of the 78 stimulation experiments met the behavioral criteria and were used in subsequent analyses.

### Neuronal analysis

We recorded 96 neurons in the intermediate and deeper layer of SC. For the saccade foraging task, the peri-stimulus time histograms (bin width 30 ms, 15 ms steps) were aligned on the beginning of fixation, reward delivery and saccade onset. Each neuron’s activity was normalized by the average neuronal responses during the last 300 ms of fixation period (300 ms before reward delivery) when a valued target was in the RF. We divided neuronal activities by the normalization factor to get the normalized response for each neuron. If the normalization factor was <5 spikes/s, the neuron was defined as unmodulated by the task (*N* = 12). A total of 53 experiments (monkey D, 24; monkey R, 29) satisfied the behavioral and efficiency criteria outlined above and were sufficiently modulated by the task to be included in further neuronal analyses.

#### General linear regression analysis

We generated a general multiple linear regression model to assess the contribution of value and choice direction on the neuronal responses (Fig. 4b). Only the first and second value rankings were used in the menu of 3-values remaining condition because monkeys rarely chose the 3rd value ranking targets. When only 3rd value ranking targets remained, all the targets were associated with the same value, therefore only the factor of choice direction was used.

We used the following regression model to fit the neuronal responses:

8 $${\rm{FR}}(t)=b0(t)+b1(t)*{\rm{Value}}+b2(t)* {\rm{Choice}}$$

Where FR represents the firing rate, *t* represents the time, Value is the value ranking (either 1 or 0 representing the higher or lower value ranking, respectively) and Choice is the direction of the next saccade (either 1 or 0 depending on whether the next saccade was directed into or out of the response field, respectively). The analyses show the impact on FR when the level of each factor changes. Statistical significance was determined with a *t* test with a false-discovery rate (FDR) correction^50^.

#### Receiver-operating characteristic (ROC) analysis

ROC analysis was used at each time point to investigate how the decision process evolved for each value ranking^51,52^. ROC curves were derived from the distributions of choice-in activities and choice-out activities of the same value ranking within a given menu (Fig. 4c). For Fig. 6, ROC curves were derived from the distributions of choice-in activities of different value ranking and choice-out activities of the highest value ranking within a given menu.

### Electrical stimulation experiments

The parameters for electrical stimulation were determined using the delayed saccade task. Clear delay-period multi-unit activity must be detected before stimulation to ensure stimulation sites were comparable to recording sites. To find the stimulation threshold, 0.25 ms biphasic currents (10-ms duration, 300 Hz) were applied during the delay period and systematically reduced from 30 μA until only hypometric saccades could be triggered. We set this intensity as the threshold intensity (monkey R, average 13.6 μA; monkey D, average 19.3 μA). During the stimulation saccade foraging task, we increased the stimulation duration to 300 ms and decreased the frequency to 150 Hz.

Sub-threshold stimulation was randomly applied on half of the fixations on 2/3 of the trials. The remaining 1/3 of trials were control trials. Stimulation trials and control trials were randomly interleaved. Stimulation was applied in the last 300 ms of the fixation period before reward delivery. The stimulation bias was the proportion of saccades directed toward the stimulation site for stimulation trials minus non-stimulation trials in every block.

### General statistical analysis

The normality of data distributions was tested for comparison between two or three groups. For n-way ANOVA analyses, data in all the conditions were assumed to be normal.

### Reporting summary

Further information on research design is available in the Nature Research Reporting Summary linked to this article.

## Supplementary information

Supplementary Information

Peer Review File

Description of Additional Supplementary Files

Supplementary Movie 1

Reporting Summary

## Data Availability

Source data are provided with this paper. A portion of the data is available on Mendeley: https://data.mendeley.com/datasets/fbmpddkbd7/1. Full data are available from the corresponding author upon reasonable request. Source data are provided with this paper.

## Source data

Source Data

## Code availability

The MatLab codes used to analyze the data contained in this study have been deposited in Mendeley: https://data.mendeley.com/datasets/fbmpddkbd7/1.
