# Supplementary material for: Transforming absolute value to categorical choice in primate superior colliculus during value-based decision making
Source: Nat Commun. 2021 Jun 7;12:3410. doi: 10.1038/s41467-021-23747-z (PMC8184840; doi:10.1038/s41467-021-23747-z)
Supplement: Supplementary file 1 — Supplementary Information [file 41467_2021_23747_MOESM1_ESM.pdf]

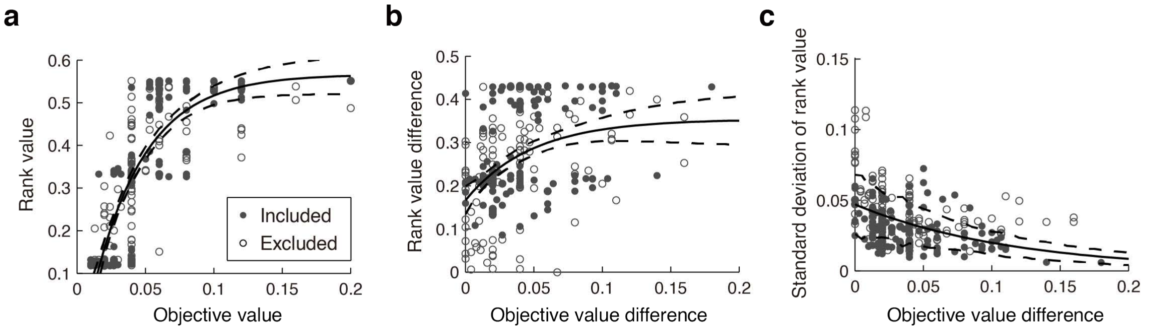

Supplementary Figure 1 The relationship between rank value and objective value across experiments. Best-fitting exponential functions plotted with 95% confidence interval. (a) Rank value increased with objective value. Adjusted $R^2 = 0.67$. (b) Differences between rank values increased with difference between objective values. Adjust $R^2 = 0.18$. (c) In each block, the average standard deviation of rank values from two colors decreased with difference between their objective values. Adjust $R^2 = 0.15$. For b and c, on the x-axis the absolute value of the difference between objective value of Rank1 and Rank2, Rank1 and Rank3, Rank2 and Rank3 were calculated. For the y-axis, the corresponding absolute value of the difference between rank values of Rank1 and Rank2, Rank1 and Rank3 and Rank2 and Rank3 were calculated. Thus, each session contributes 3 data points. (filled dots, included in subsequent neuronal analyses; unfilled dots, excluded from further neuronal analyses)

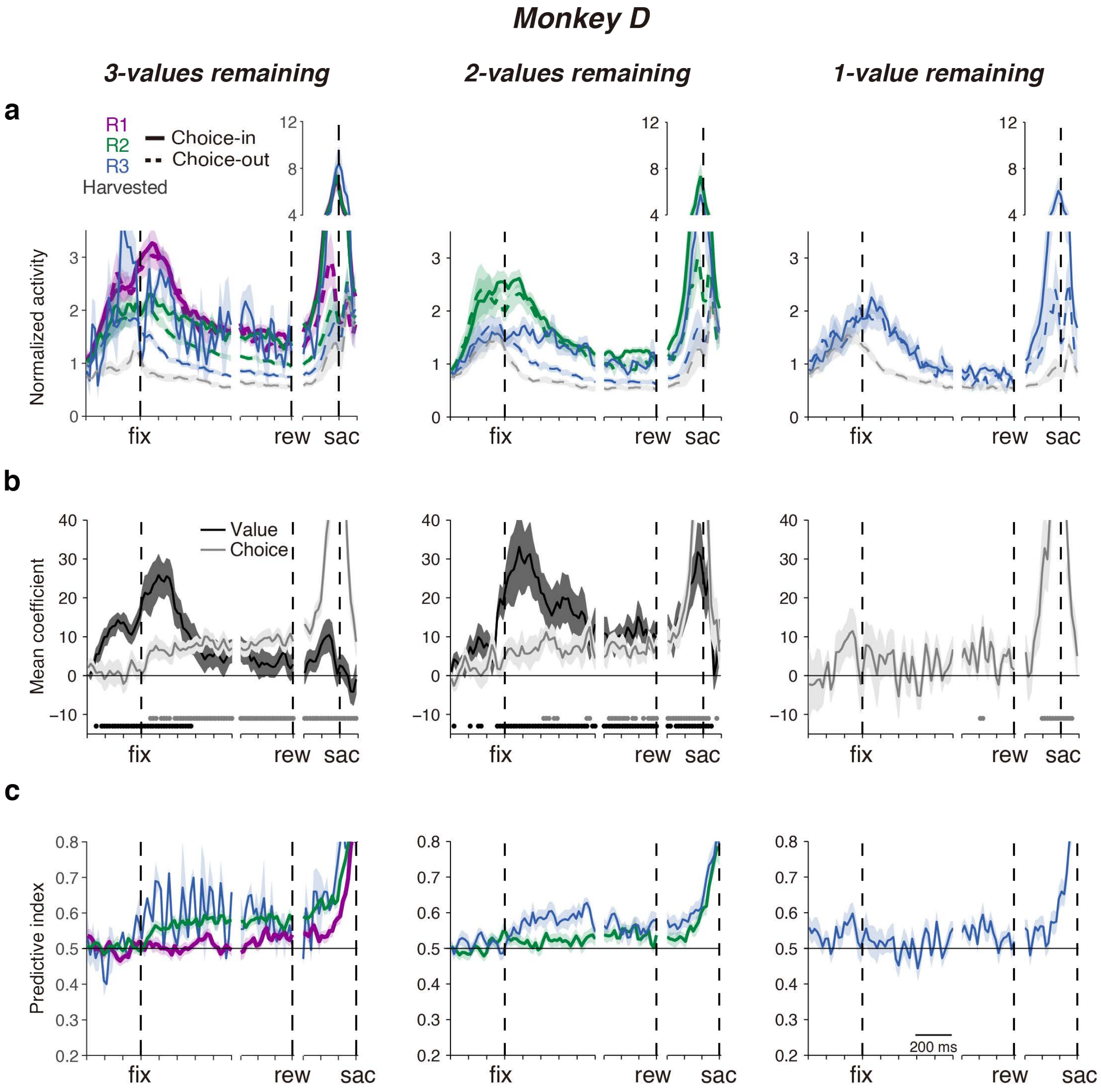

Supplementary Figure 2 The evolution of value and choice signals in SC activity across 3 menus from monkey D. N = 24. Same format as **Fig. 4**.

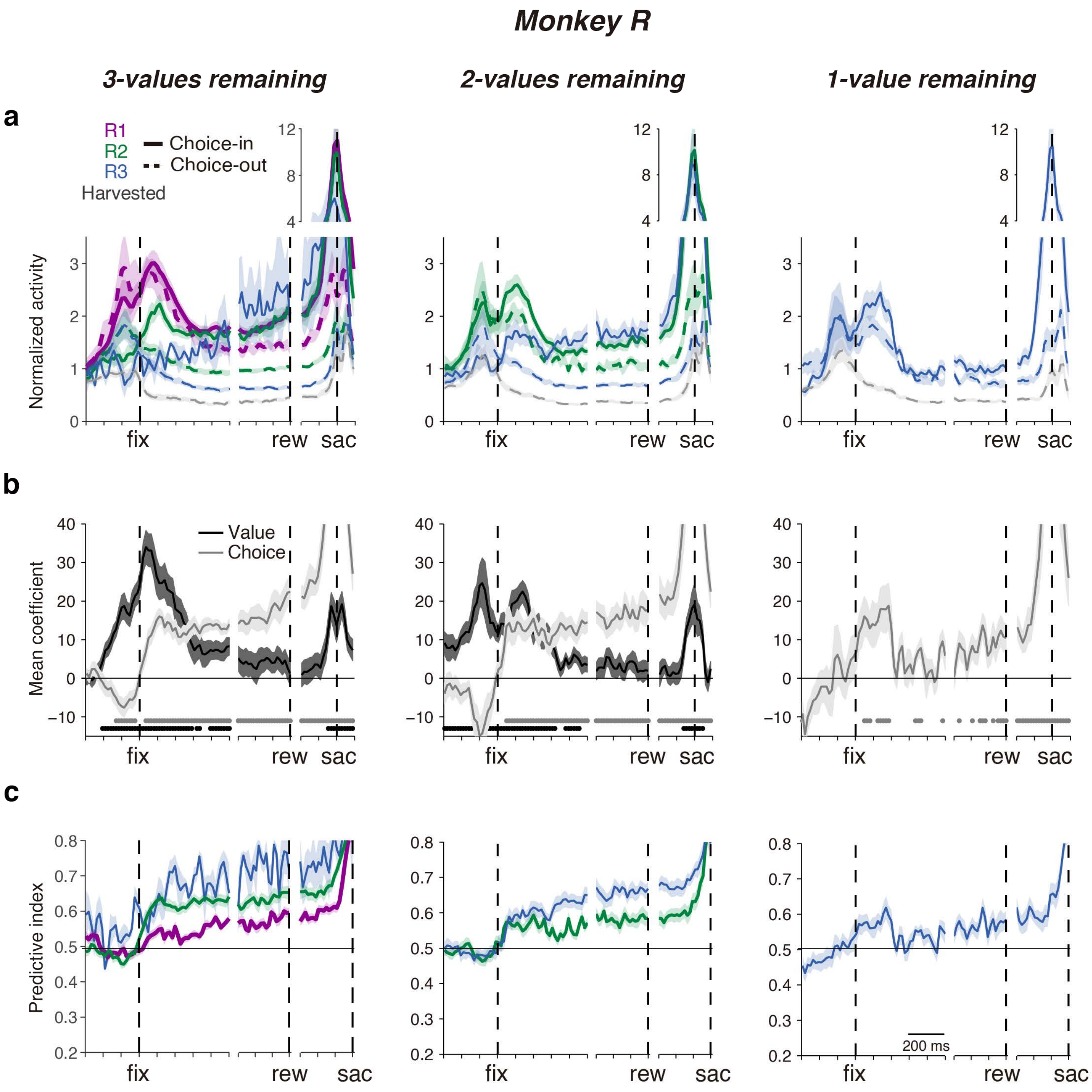

Supplementary Figure 3 The evolution of value and choice signals in SC activity across 3 menus from monkey R. N = 29. Same format as **Fig. 4**.

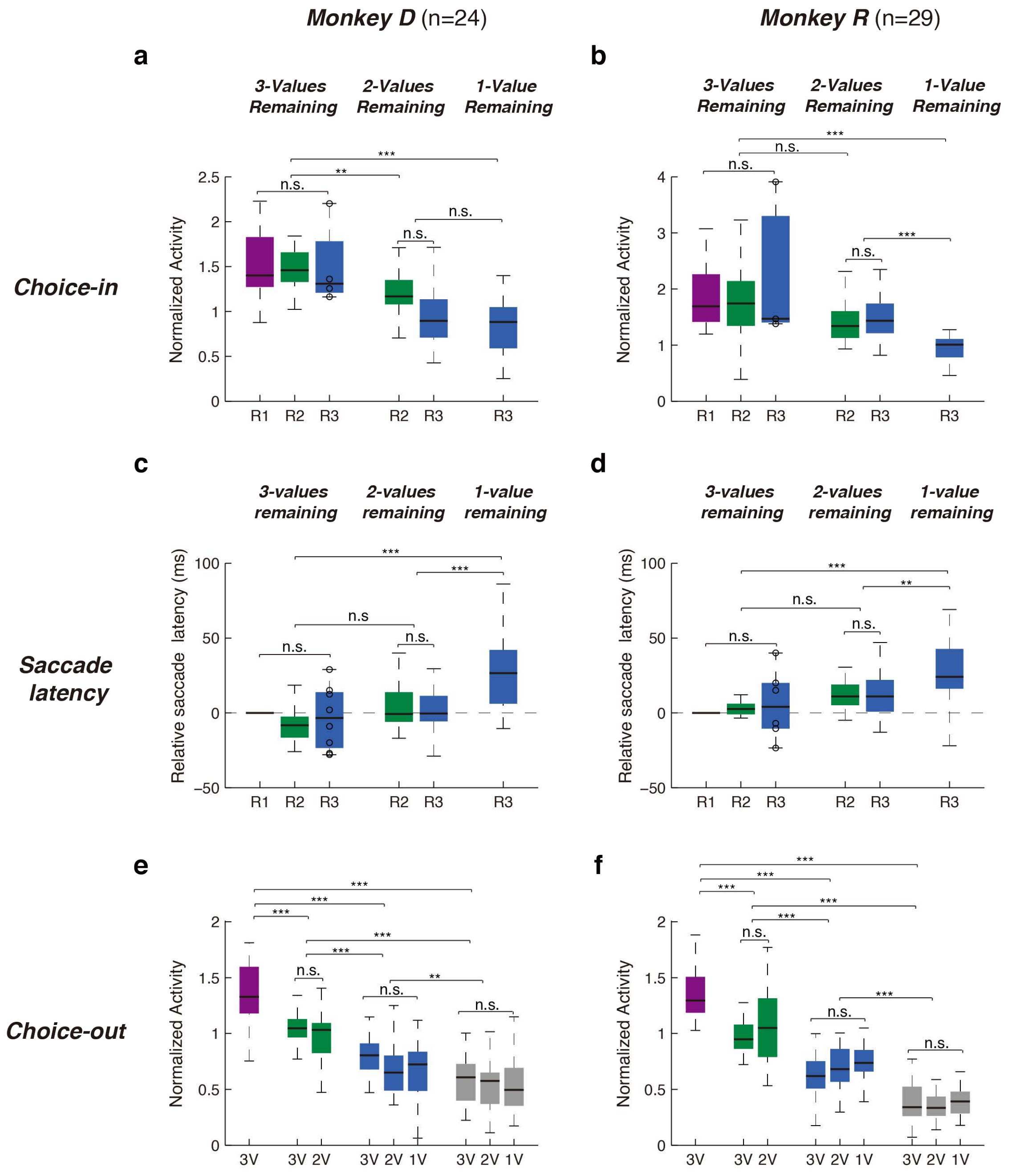

Supplementary Figure 4 Menu updating of the value-threshold level and value representations in the late fixation period for individual monkeys. Monkey D (a,c,e) and monkey R (b,d,f), respectively. Same format as **Fig. 5**. For conditions from left to right (a) n = 24, 23, 4, 24, 18 and 22; (b) n = 29, 28, 3, 29, 26 and 25; (c) n = 24, 24, 8, 24, 16 and 24; (d) n =29, 26, 6, 29, 24 and 29; (e) n = 20, 24, 21, 24, 24, 16, 24, 24 and 24; (f) n = 29, 29, 28, 29, 29, 23, 29, 29 and 29. n in (a-f) represents the number of blocks with more than 3 cases for the condition. N-way ANOVA tests, post hoc tests were done with Bonferroni correction, the test results (labelled with any number of *) from top to down (a) P = 2.8×10^{-4}, 0.0013; (b) P = 1.2×10^{-5}, 3.3×10^{-4}; (c) P = 2.0×10^{-7}, 9.5×10^{-6}; (d) P = 3.8×10^{-4}, 0.0089; (e) P = 1.5×10^{-21}, 2.7×10^{-15}, 4.2×10^{-5}, 1.2×10^{-14}, 3.0×10^{-7}, 4.3×10^{-3}; (f) P = 7.0×10^{-48}, 1.2×10^{-29}, 1.6×10^{-10}, 2.6×10^{-35}, 6.6×10^{-13}, 1.7×10^{-13}. For the boxplots, on each box, the central mark is the

median, the edges of the box are the 25th and 75th percentiles, and the whiskers extend to the most extreme data points that the algorithm considers not to be outliers. Outliers are data points that are larger than Q3 + 1.5 × (Q3 – Q1) or smaller than Q1 – 1.5 × (Q3 – Q1), where Q1 and Q3 are the 25th and 75th percentiles, respectively. Source data are provided as a Source Data file. (n.s., nonsignificant, *P < 0.05, **P < 0.01, ***P < 0.001;).

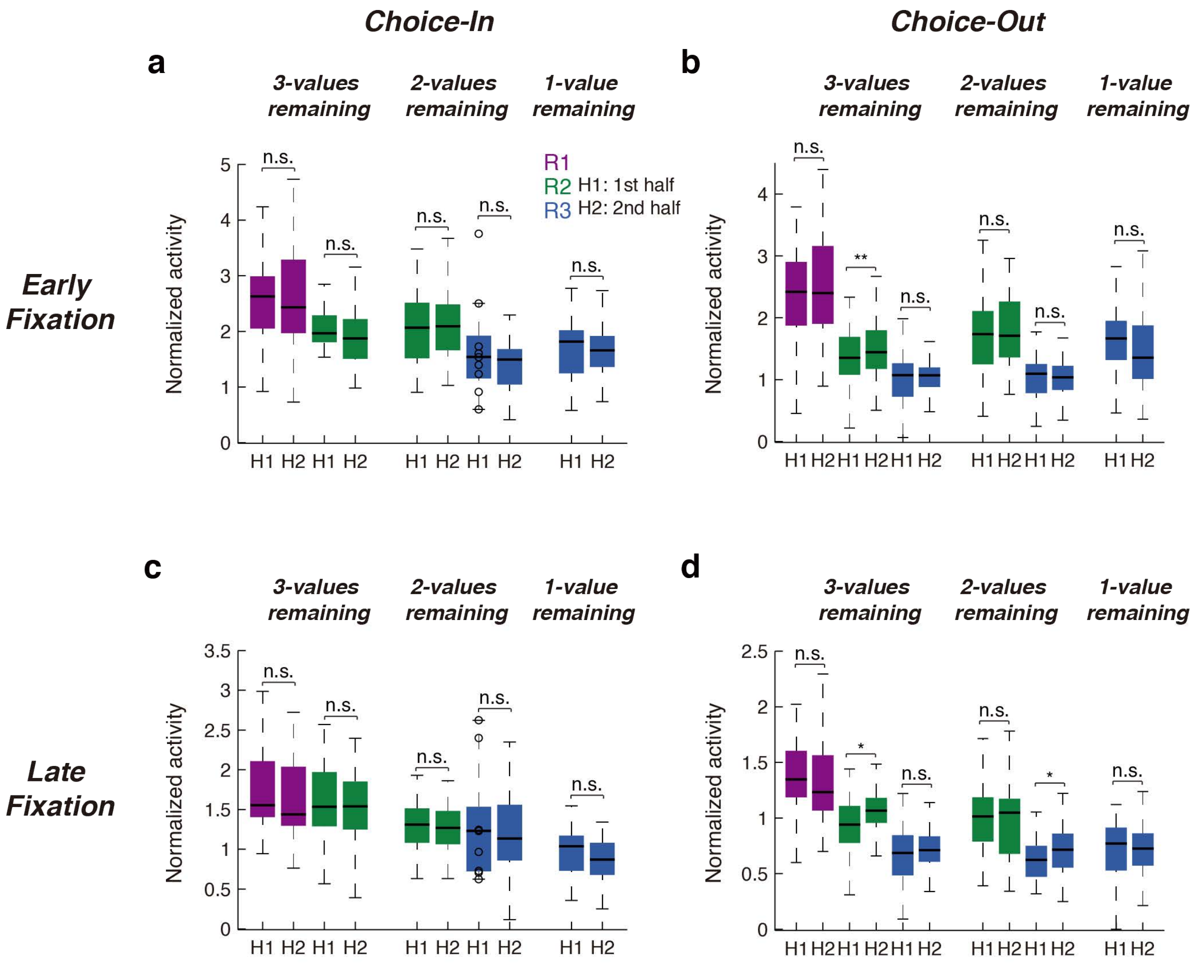

Supplementary Figure 5 Menu transitions, not number of remaining targets, influenced value-threshold level activity. Data were compared between first half (H1) and second half (H2) choices in a menu. Early fixation period: (a) Choice-In condition. For conditions from left to right n = 53, 53, 22, 52, 53, 53, 9, 47, 26 and 47. The third value ranking in 3-values menu is absent due to lack of data. (b) Choice-Out condition. For conditions from left to right n = 49, 35, 53, 53, 53, 53, 48, 36, 53, 53, 23 and 32. Late fixation period: (c) Choice-In condition. n is same as (a). (d) Choice-Out condition. n is same as (b). n in (a-d) represents the number of blocks with more than 3 cases for the condition. Wilcoxon signed rank tests, two-sided, with False Discovery Rate correction, the test results (labelled with any number of *) from left to right (b) P = 0.0033; (d) P = 0.018, 0.013. For the boxplots, on each box, the central mark is the median, the edges of the box are the 25th and 75th percentiles, and the whiskers extend to the most extreme data points that the algorithm considers not to be outliers. Outliers are data points that are larger than Q3 + 1.5 × (Q3 – Q1) or smaller than Q1 – 1.5 × (Q3 – Q1), where Q1 and Q3 are the 25th and 75th percentiles, respectively. Source data are provided as a Source Data file. (n.s. nonsignificant, *P < 0.05, **P < 0.01, ***P < 0.001)

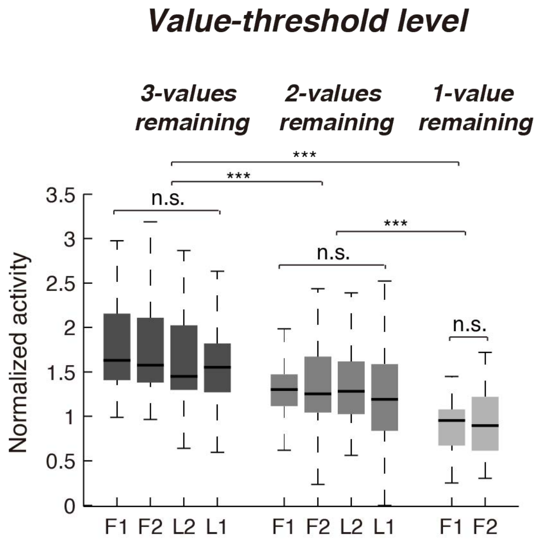

Supplementary Figure 6 Value-threshold level decreased immediately after menu changed**.** (F1, F2: first and second choice within a menu; L2, L1: the penultimate and last choice within a menu). Here we did not segregate based on value of the items because the value-threshold level was the same regardless of value. For conditions from left to right n = 53, 53, 53, 51, 53, 53, 53, 53, 51 and 42. n represents the number of blocks with more than 3 cases for the condition. N-way ANOVA test, post hoc tests were done with Bonferroni correction, the test results (labelled with any number of *) from top to down $P = 1.1 \times 10^{-21}$, 1.1×10^{-10}, 7.7×10^{-7}. For the boxplots, on each box, the central mark is the median, the edges of the box are the 25th and 75th percentiles, and the whiskers extend to the most extreme data points that the algorithm considers not to be outliers. Outliers are data points that are larger than Q3 + 1.5 × (Q3 – Q1) or smaller than Q1 – 1.5 × (Q3 – Q1), where Q1 and Q3 are the 25th and 75th percentiles, respectively. Source data are provided as a Source Data file. (n.s. nonsignificant, *$P < 0.05$, **$P < 0.01$, ***$P < 0.001$)

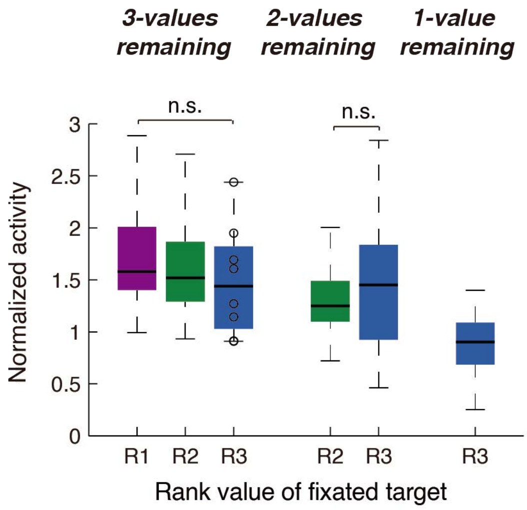

Supplementary Figure 7 Value of fixated target does not affect value-threshold level. The value of the target being currently fixated did not affect the value-threshold level within a given menu. Data is presented as a function of the value of the fixated target when there were three values, two values or one value targets remaining in the array and the monkey ultimately chose the target in the RF. In this case, we are examining the value of the fixated target regardless of the value of the target in the RF. R1 denotes current fixation of the highest rank value target, R2 for middle rank value target and R3 for lowest rank value target. For conditions from left to right n = 53, 41, 8, 53, 23 and 49. n represents the number of blocks with more than 3 cases for the condition. For the boxplots, on each box, the central mark is the median, the edges of the box are the 25th and 75th percentiles, and the whiskers extend to the most extreme data points that the algorithm considers not to be outliers. Outliers are data points that are larger than Q3 + 1.5 × (Q3 – Q1) or smaller than Q1 – 1.5 × (Q3 – Q1), where Q1 and Q3 are the 25th and 75th percentiles, respectively. Source data are provided as a Source Data file. (Kruskal-Wallis test and Wilcoxon signed rank test, two-sided; n.s. nonsignificant)

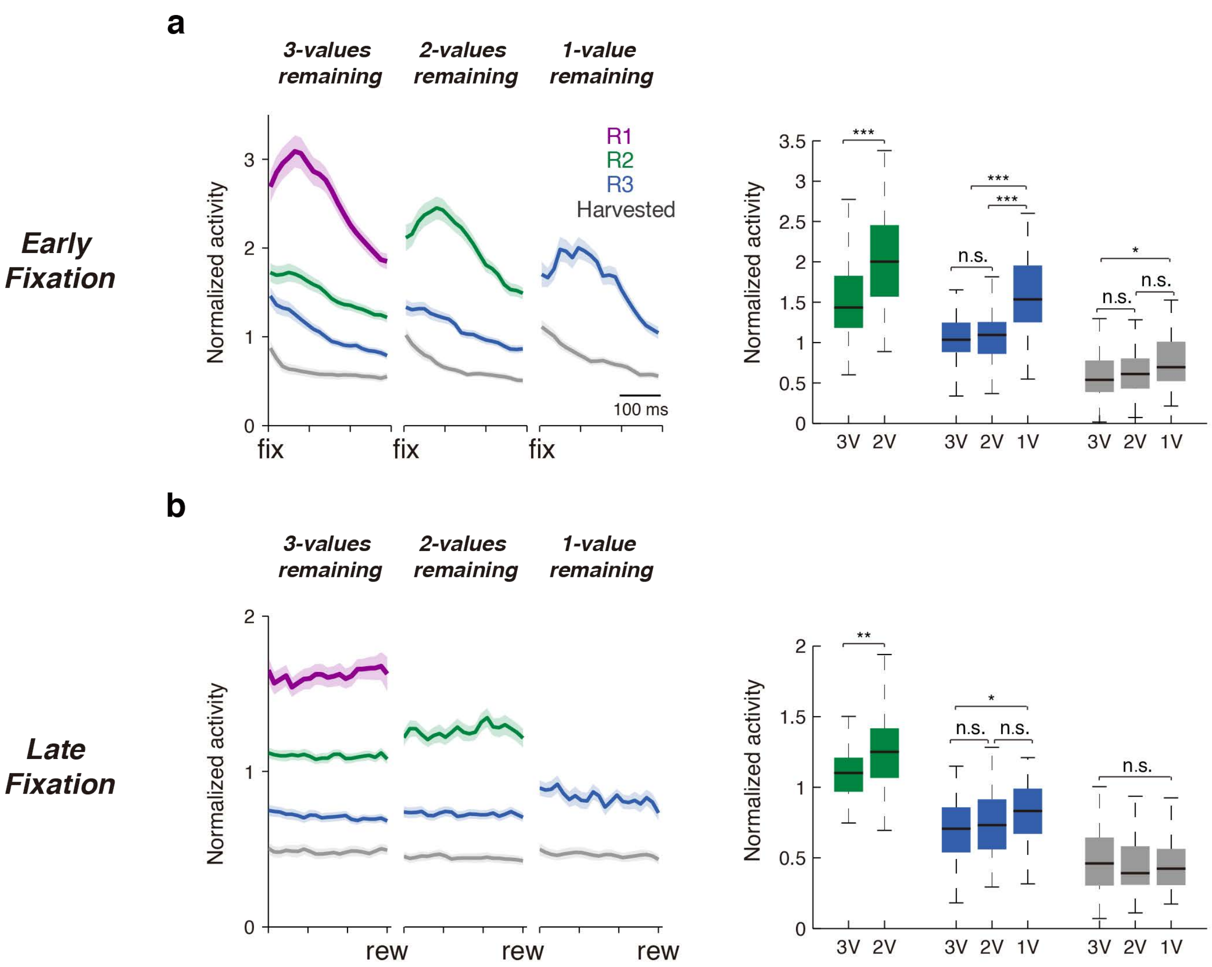

Supplementary Figure 8 SC activity related to the value of the target in the response field was modulated by menu. Same data as **Fig. 3**. Data is presented for each value ranking from early fixation period (a) and late fixation period (b). For both conditions from left to right n = 53, 53, 53, 53, 52, 53, 53 and 53. n in (a, b) represents the number of blocks with more than 3 cases for the condition. Two-sided Paired-t tests and one-way ANOVA tests, post hoc tests were done with Bonferroni correction, the test results (labelled with any number of *) from top to down (a) $P = 1.1 \times 10^{-12}$, 2.5×10^{-10}, 3.5×10^{-9}, 0.031; (b) P = 0.0029, 0.020. For the boxplots, on each box, the central mark is the median, the edges of the box are the 25th and 75th percentiles, and the whiskers extend to the most extreme data points that the algorithm considers not to be outliers. Outliers are data points that are larger than $Q3 + 1.5 \times (Q3 - Q1)$ or smaller than $Q1 - 1.5 \times (Q3 - Q1)$, where Q1 and Q3 are the 25th and 75th percentiles, respectively. Source data are provided as a Source Data file. (n.s., nonsignificant, $*P < 0.05$, $**P < 0.01$, $***P < 0.001$)

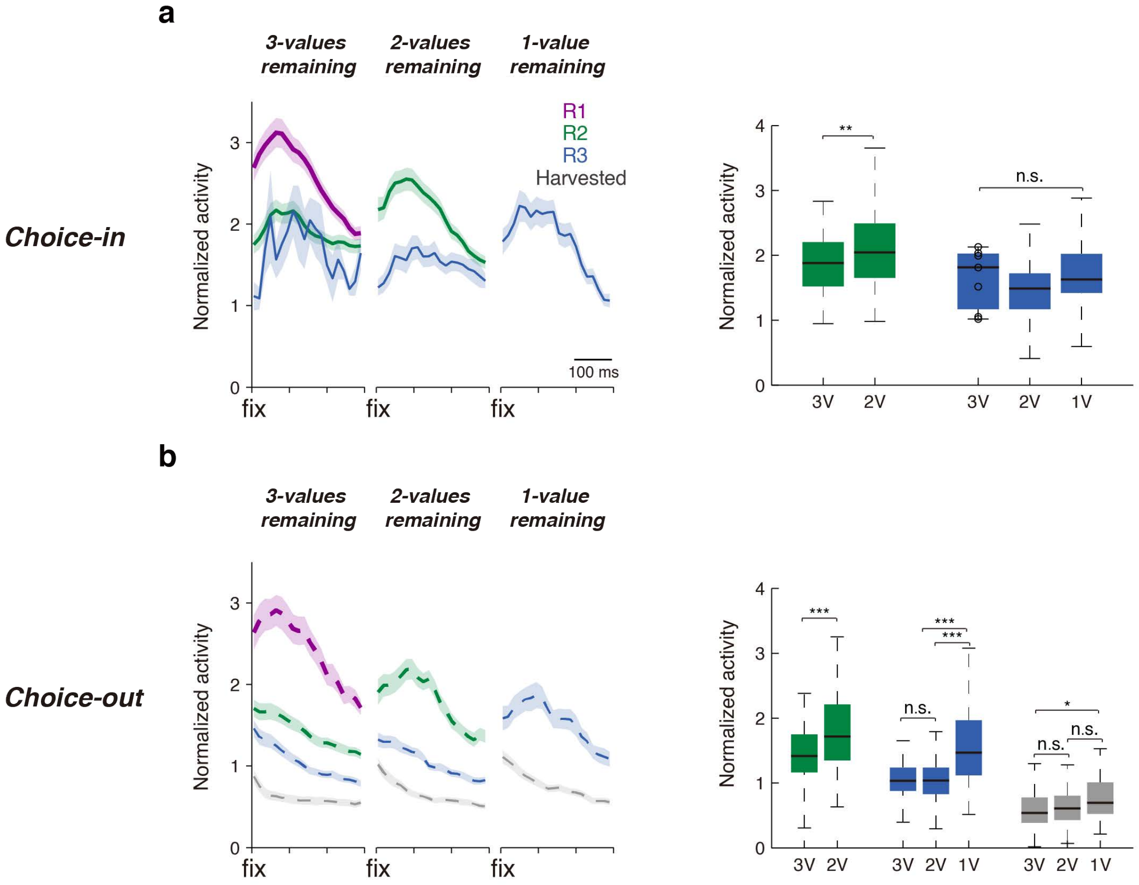

Supplementary Figure 9 Menu-dependent value activity during early fixation. Normalized neuronal activity in the first 300 ms of the fixation time from choice-in (a) and choice-out conditions (b). Same format as **Fig. 5**. Data is presented for each value ranking when there were three values (3V), two values (2V) or one value (1V) targets remaining in the array. For choice-in conditions from left to right n = 51, 53, 7, 44 and 47. For choice-out conditions from left to right n = 53, 49, 53, 53, 39, 53, 53 and 53. n in (a, b) represents the number of blocks with more than 3 cases for the condition. Two-sided Paired-t tests and one-way ANOVA tests, post hoc tests were done with Bonferroni correction, the test results (labelled with any number of *) from left to right (a) $P = 0.0024$; (b) $P = 8.0 \times 10^{-9}$, 7.8×10^{-7}, 1.1×10^{-6}, 0.029. For the boxplots, on each box, the central mark is the median, the edges of the box are the 25th and 75th percentiles, and the whiskers extend to the most extreme data points that the algorithm considers not to be outliers. Outliers are data points that are larger than $Q3 + 1.5 \times (Q3 - Q1)$ or smaller than $Q1 - 1.5 \times (Q3 - Q1)$, where Q1 and Q3 are the 25th and 75th percentiles, respectively. Source data are provided as a Source Data file. (n.s., nonsignificant, $^{*}P < 0.05$, $^{**}P < 0.01$, $^{***}P < 0.001$)

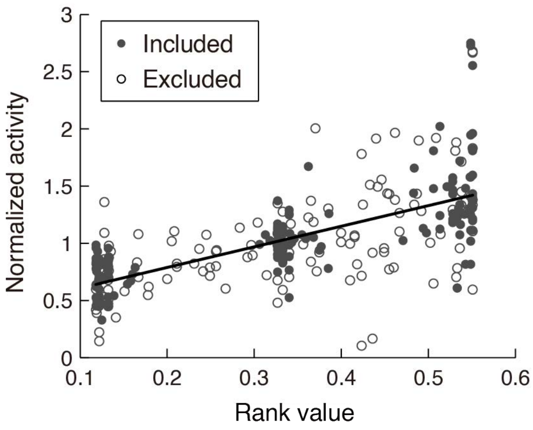

Supplementary Figure 10 Choice-out SC activity during the late fixation period (300 ms before reward delivery) was correlated with RF target rank value. As the value coding was invariant with menu, neuronal activity was collapsed across all menus. In total, 288 data points were derived from 96 blocks with 3 target values each. To provide a fuller range of rank values for this correlation, data from previous analyses were included (filled circles) as well as data excluded (unfilled circles) from previous analyses because of unstable preferences over experimental sessions and/or low late fixation period neuronal activity. Pearson correlation coefficients: $R^2 = 0.44$, $P = 7.2 \times 10^{-38}$.

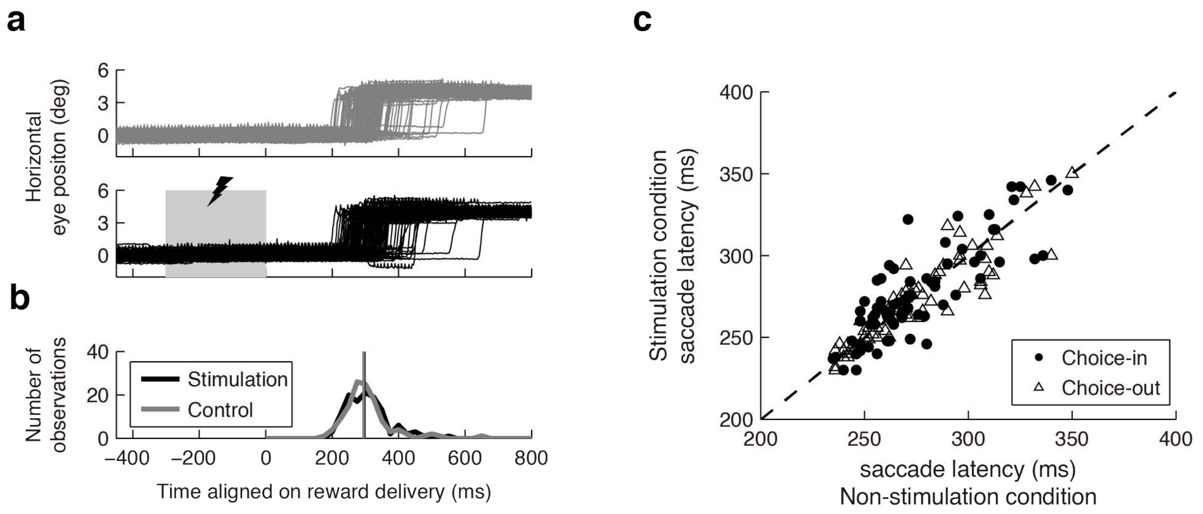

Supplementary Figure 11 Micro-stimulation was sub-threshold for directly triggering saccades. (a) Horizontal eye position for saccades directed towards the stimulation site for an example block of trials. Gray traces denote the non-stimulation, control condition and black traces denote the stimulation condition. The shaded gray area and associated lightning bolt indicate the period during which micro-stimulation was applied. The time is aligned on reward delivery. (b) The corresponding saccade latency distributions for an example block. Vertical solid lines indicate the median latency of the distributions. (c) Median saccade latency for each stimulation site during stimulation compared with non-stimulation condition. Choices toward the stimulation site are denoted by circles and choices out of the stimulation sites are denoted by triangles. All the 72 blocks in stimulation experiments were included. (Two-sided, Wilcoxon signed rank test, Choice-in, P = 0.36; Choice-out, P = 0.74.)

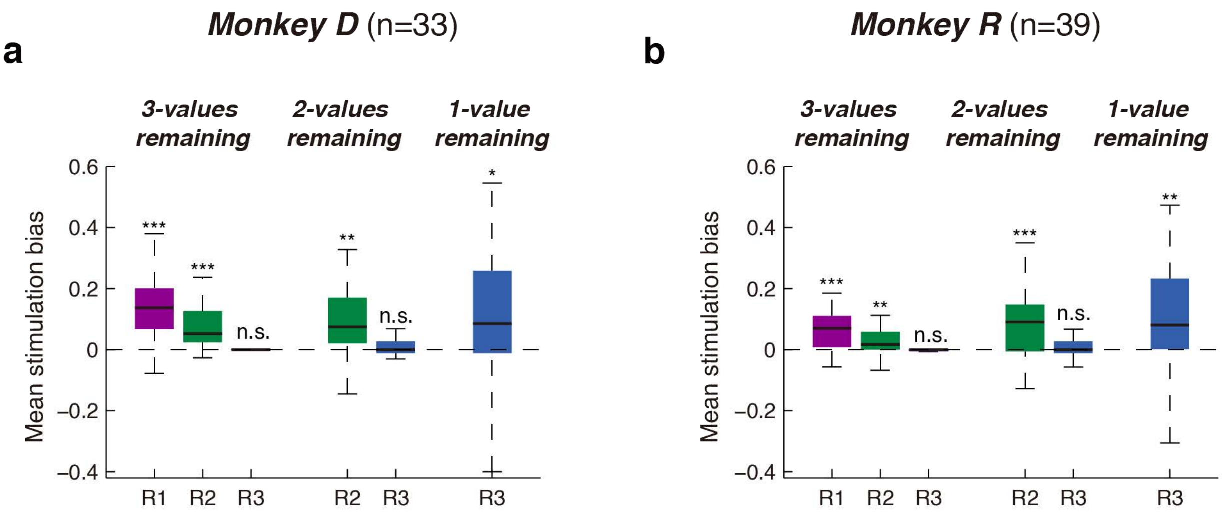

Supplementary Figure 12 Electrical micro-stimulation results from individual monkeys. Same format as **Fig. 7**. (a) Data from monkey D. (b) Data from monkey R. All blocks in stimulation experiments were included. Wilcoxon signed rank test, two-sided, from left to right, (a) $P = 2.3 \times 10^{-6}$, 2.7×10^{-6}, 0.55, 0.0029, 0.41 and 0.010; (b) $P = 4.3 \times 10^{-5}$, 0.0071, 0.47, 2.2×10^{-4}, 0.53 and 0.0032. For the boxplots, on each box, the central mark is the median, the edges of the box are the 25th and 75th percentiles, and the whiskers extend to the most extreme data points that the algorithm considers not to be outliers. Outliers are data points that are larger than $Q3 + 1.5 \times (Q3 - Q1)$ or smaller than $Q1 - 1.5 \times (Q3 - Q1)$, where Q1 and Q3 are the 25th and 75th percentiles, respectively. Source data are provided as a Source Data file. (n.s., nonsignificant, $*P < 0.05$, $**P < 0.01$, $***P < 0.001$)

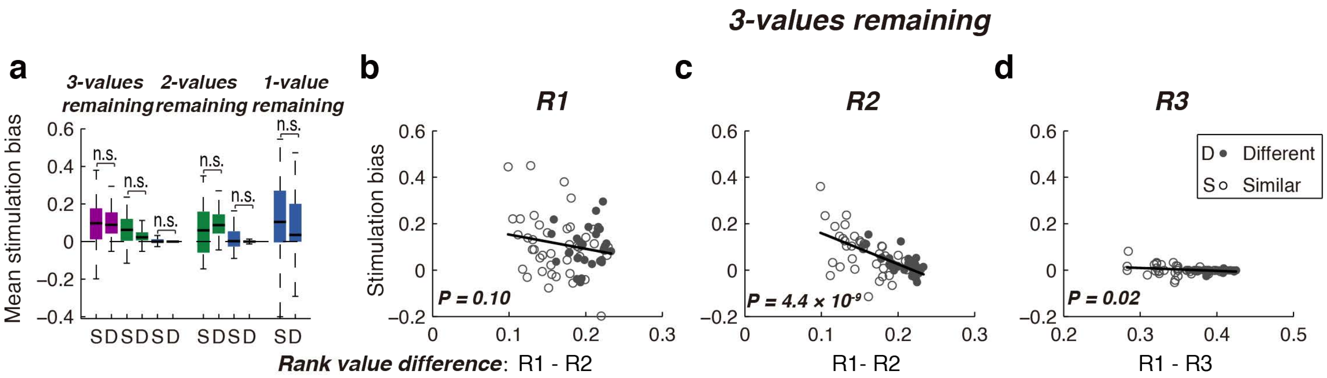

Supplementary Figure 13 Same format as **Fig. 7**. Simulation bias effect in Similar-value (S) blocks (n = 40) versus Different-value (D) blocks (n = 32) in stimulation experiments. See Methods for details. (a) Average bias effect across blocks. (Two-sided, Wilcoxon rank sum test) Source data are provided as a Source Data file. (b-d) The relationship between bias effect and difference of rank values. (b,c) In menu of 3-values remaining, the stimulation bias effect on choosing Rank1 (b) and Rank2 (c) is plot against the difference between rank value of Rank1 and Rank2. Pearson correlation coefficients: R = -0.20 (b) and R = -0.63 (c). (d) In menu of 3-values remaining, the stimulation bias effect on choosing Rank3 is plot against the difference between rank value of Rank1 and Rank3. Pearson correlation coefficients: R = -0.27. For the boxplots, on each box, the central mark is the median, the edges of the box are the 25th and 75th percentiles, and the whiskers extend to the most extreme data points that the algorithm considers not to be outliers. Outliers are data points that are larger than Q3 + 1.5 ×(Q3 – Q1) or smaller than Q1 – 1.5 ×(Q3 – Q1), where Q1 and Q3 are the 25th and 75th percentiles, respectively.

Supplementary Table 1 Possible target values during neuronal recordings

Value (μL/s)		Fixation time (s)				
		**0.5**	**1**	**1.5**	**2**	**2.5**
Reward magnitude (μL)	**20**	40	20	13	10	8
	**40**	80	40	27	20	16
	**60**	120	60	40	30	24
	**80**	160	80	53	40	32
	**100**	200	100	67	50	40
