# Supplementary material for: Transforming absolute value to categorical choice in primate superior colliculus during value-based decision making
Source: Nat Commun. 2021 Jun 7;12:3410. doi: 10.1038/s41467-021-23747-z (PMC8184840; doi:10.1038/s41467-021-23747-z)
Supplement: Supplementary file 3 — Description of Additional Supplementary Files [file 41467_2021_23747_MOESM3_ESM.docx]

Description of Additional Supplementary Files

Title: Supplementary Movie 1

Description: Movie for example trial of saccade foraging task (same trial as Fig. 2a). The white dot represents foveal eye position. The gray circle denotes the location and approximate size of the SC neuronal response field that is represented in retinotopic coordinates and consequentially moves in concert with the eye. The auditory beeps represent the timing and duration of liquid reward delivery. The timing of neuronal spikes is represented by the white raster and associated auditory clicks. The duration of fixation and mean firing rate associated with different colored targets in the neuron’s response field are denoted by the vertical and horizontal colored bars at the bottom, respectively.
