# Supplementary material for: Transforming absolute value to categorical choice in primate superior colliculus during value-based decision making
Source: Nat Commun. 2021 Jun 7;12:3410. doi: 10.1038/s41467-021-23747-z (PMC8184840; doi:10.1038/s41467-021-23747-z)
Supplement: Supplementary file 5 — Reporting Summary [file 41467_2021_23747_MOESM5_ESM.pdf]

nature research

Corresponding author(s): Beizhen Zhang and Michael Christopher Dorris

Last updated by author(s): Apr 21, 2021

Reporting Summary

Nature Research wishes to improve the reproducibility of the work that we publish. This form provides structure for consistency and transparency in reporting. For further information on Nature Research policies, see our Editorial Policies and the Editorial Policy Checklist.

Statistics

For all statistical analyses, confirm that the following items are present in the figure legend, table legend, main text, or Methods section.

n/a	Confirmed	
☐	☒	The exact sample size (*n*) for each experimental group/condition, given as a discrete number and unit of measurement
☐	☒	A statement on whether measurements were taken from distinct samples or whether the same sample was measured repeatedly
☐	☒	The statistical test(s) used AND whether they are one- or two-sided<br>*Only common tests should be described solely by name; describe more complex techniques in the Methods section.*
☒	☐	A description of all covariates tested
☐	☒	A description of any assumptions or corrections, such as tests of normality and adjustment for multiple comparisons
☐	☒	A full description of the statistical parameters including central tendency (e.g. means) or other basic estimates (e.g. regression coefficient) AND variation (e.g. standard deviation) or associated estimates of uncertainty (e.g. confidence intervals)
☐	☒	For null hypothesis testing, the test statistic (e.g. *F*, *t*, *r*) with confidence intervals, effect sizes, degrees of freedom and *P* value noted<br>*Give P values as exact values whenever suitable.*
☒	☐	For Bayesian analysis, information on the choice of priors and Markov chain Monte Carlo settings
☒	☐	For hierarchical and complex designs, identification of the appropriate level for tests and full reporting of outcomes
☐	☒	Estimates of effect sizes (e.g. Cohen's *d*, Pearson's *r*), indicating how they were calculated

Our web collection on statistics for biologists contains articles on many of the points above.

Software and code

Policy information about availability of computer code

Data collection	Data collection is stated in the manuscript as follows: The position of the left eye was sampled at 500 Hz by an EYELINK1000 infrared eye tracker (SR Research). Behavioral tasks were under the control of Monkeylogic software (http://www.monkeylogic.net/). Single-cell discharges were sampled at 22 kHz using the AlphaLab SnR System and subsequently offline sorted using Spike 2 (version 7.15; CED, Cambridge Electronic Design Limited).
Data analysis	All data analyses were performed offline using custom built code in MATLAB (version R2014a, Mathworks Inc). The MatLab codes used to analyze the data contained in this study have been deposited in Mendeley: https://data.mendeley.com/datasets/fbmpddkbd7/1.

For manuscripts utilizing custom algorithms or software that are central to the research but not yet described in published literature, software must be made available to editors and reviewers. We strongly encourage code deposition in a community repository (e.g. GitHub). See the Nature Research guidelines for submitting code & software for further information.

Data

Policy information about availability of data

All manuscripts must include a data availability statement. This statement should provide the following information, where applicable:

- Accession codes, unique identifiers, or web links for publicly available datasets
- A list of figures that have associated raw data
- A description of any restrictions on data availability

Source data are provided with this paper. A portion of the data is available on Mendeley: https://data.mendeley.com/datasets/fbmpddkbd7/1. Full data are available from the corresponding author upon reasonable request.

Field-specific reporting

Please select the one below that is the best fit for your research. If you are not sure, read the appropriate sections before making your selection.

- [x] Life sciences
- [ ] Behavioural & social sciences
- [ ] Ecological, evolutionary & environmental sciences

For a reference copy of the document with all sections, see nature.com/documents/nr-reporting-summary-flat.pdf

Life sciences study design

All studies must disclose on these points even when the disclosure is negative.

Sample size	Two experimental subjects (rhesus monkeys) were used in this study. This number is typical for non-human primate electrophysiology studies. The total number of neurons (n=96) and microstimulation sites (n=72) were determined by the limits of data collection within a reasonable time, and were compared with references cited.
Data exclusions	We excluded some trials in recording and stimulation experiments, if they did not meet the behavioral criteria. We also excluded 12 neurons in recording experiments if their firing rate was very low (less than 5 spikes/s) during the task. (Methods)
Replication	Results in behavioral performance, recording and stimulation experiments were consistent across two rhesus monkeys.
Randomization	Once a Superior Colliculus neuron was isolated or appropriate Superior Colliculus microstimulation site identified, all experimental protocols were automatically randomized in the MonkeyLogic software. This included such parameters as randomized trial types, target locations, target colour/value associations, and application of electrical microstimulation. The randomization of all these parameters are clearly described in the manuscript.
Blinding	The group allocation was not predetermined , but was separated with code from monkey's choice strategy. The behavioral responses were objectively measured by automated hardware and software. Thus, there was no blinding in this study.

Reporting for specific materials, systems and methods

We require information from authors about some types of materials, experimental systems and methods used in many studies. Here, indicate whether each material, system or method listed is relevant to your study. If you are not sure if a list item applies to your research, read the appropriate section before selecting a response.

Materials & experimental systems

n/a	Involved in the study	
☒	☐	Antibodies
☒	☐	Eukaryotic cell lines
☒	☐	Palaeontology and archaeology
☐	☒	Animals and other organisms
☒	☐	Human research participants
☒	☐	Clinical data
☒	☐	Dual use research of concern

Methods

n/a	Involved in the study	
☒	☐	ChIP-seq
☒	☐	Flow cytometry
☒	☐	MRI-based neuroimaging

Animals and other organisms

Policy information about studies involving animals; ARRIVE guidelines recommended for reporting animal research

Laboratory animals	Two male rhesus monkeys (Macaca mulatta; D, 12 kg, and R, 8 kg) participated in this study. Ages were between 5-7 years old.
Wild animals	This study did not involve wild animals.
Field-collected samples	This study did not involve data collected from field samples.
Ethics oversight	All animal use procedures were approved by the Animal Care and Use Committee of the Institute of Neuroscience, Chinese Academy of Sciences.

Note that full information on the approval of the study protocol must also be provided in the manuscript.
