# Supplementary material for: Transforming absolute value to categorical choice in primate superior colliculus during value-based decision making
Source: Nat Commun. 2021 Jun 7;12:3410. doi: 10.1038/s41467-021-23747-z (PMC8184840; doi:10.1038/s41467-021-23747-z)
Supplement: Supplementary file 2 — Peer Review File [file 41467_2021_23747_MOESM2_ESM.pdf]

Editorial Note: This manuscript has been previously reviewed at another journal that is not operating a transparent peer review scheme. The manuscript was considered suitable for publication without further review at Nature Communications.
